# Genetic dissection of signalling pathways that mediate iron-related tumor growth in a *Drosophila* model

**DOI:** 10.1371/journal.pgen.1012017

**Published:** 2026-02-13

**Authors:** Li Jin, Feng Gao, Ping Li, Chenxin Yu, Guiran Xiao

**Affiliations:** 1 Anhui Provincial International Science and Technology Cooperation Base for Major Metabolic Diseases and Nutritional Interventions, -Hefei University of Technology, Hefei, China; 2 School of Food and Biological Engineering, Hefei University of Technology, Hefei, China; 3 Department of Cardiology, Second Affiliated Hospital of Anhui Medical University, Hefei, China; 4 China Light Industry Key Laboratory of Meat Microbial Control and Utilization, Hefei University of Technology, Hefei, China; 5 Engineering Research Center of Bio-Process, Ministry of Education, Hefei University of Technology, Hefei, China; University of Pennsylvania, UNITED STATES OF AMERICA

## Abstract

Iron dyshomeostasis is associated with various cancers. Here we explore the underlying mechanisms through which iron promotes tumor growth and metastasis using a *Drosophila* cancer model. In this model, cells iin the eye-antennal imaginal disc co-express oncogenic Raf gain-of-function and Scribbled loss-of-function mutants, leading to tumor formation. First, we show that dietary iron overload enhances tumor growth, invasiveness and mobility of cancer cells, whereas iron chelation suppresses these phenotypes. Consistently, RNA interference (RNAi)-mediated knockdown of *dZIP13*, a zinc transporter that transports iron into the secretory pathway, results in cytosolic iron accumulation and exacerbates the cancer-like phenotypes. Second, we show that the activity of a ten-eleven translocation DNA dioxygenase (TET), which enables DNA demethylation, correlates with cellular iron bioavailability, consistent with the known requirement of iron in the catalytic site of this enzyme. Third, we show that the TET enzyme transcriptionally regulates a histone methylase responsible for the H3K27me3 epigenetic mark. Fourth, we demonstrate that the iron-dependent DNA demethylation and subsequent histone trimethylation events activate the JAK/STAT signalling pathway, which promotes tumorigenesis, including the recruitment and proliferation of hemocytes to the malignant tissue. These findings reveal a novel tumor-suppressor function for dZIP13, while providing molecular mechanisms for iron-mediated tumor progression.

## Author summary

Iron is an essential nutrient, but too much iron can be harmful and is often linked to cancer. In this study, we used fruit flies (*Drosophila*) to investigate how excess iron contributes to tumor growth. We found that altering the expression of a metal transporter called dZIP13, which controls how iron is distributed inside cells, changes the amount of iron in specific cellular compartments and affects tumor behavior. When dZIP13 function was reduced, iron accumulated in the wrong place, causing tumors to grow faster and invade nearby tissues. We also discovered that this process changes how certain genes are regulated through chemical marks on DNA and histones, activating a signalling pathway that promotes tumor growth and recruits immune-like cells to the tumor. Our work reveals how a single gene that controls iron distribution can influence gene regulation during cancer progression.

## Introduction

Iron plays a pivotal role as a structural element or a cofactor in numerous proteins that participate in enzymatic reactions [1], DNA synthesis [2], and signal transduction [3]. A lack of iron can result in the development of profound iron-deficiency anaemia along with significant metabolic disturbances, whereas excess iron can catalyze reactive oxygen species (ROS) formation through Fenton chemistry, leading to oxidative damage and cytotoxicity [4]. Therefore, iron absorption, transportation, storage, and excretion are strictly and precisely regulated by a series of proteins to maintain intracellular and systemic iron homeostasis. Iron transporters are responsible for intracellular iron storage/release and uptake/excretion and are key determinants of iron distribution and are critical regulators of iron homeostasis in all living organisms [5]. Research conducted over time has shown that disruptions in iron balance are linked to a range of health conditions, including obesity [6], neurodegenerative diseases [7], and cancer [8]. It should be highlighted that numerous investigations have confirmed a correlation between disruptions in iron homeostasis and the increased risk of cancer initiation, proliferation, and metastasis [9]. For instance, neoplastic cells display an iron-acquiring behaviour facilitated by the misregulation of proteins involved in iron metabolism during tumor development [10,11]. The levels of ferritin, a protein responsible for iron storage, are elevated and are associated with unfavourable outcomes [12]. Several studies suggest that elevated iron can modulate pathways such as WNT and HIF, thereby enhancing oxidative stress and promoting tumor growth and invasion [3]. Nevertheless, our understanding of the mechanisms that connect iron imbalance to cancer remains limited.

Human ZIP13 (ZIP13, SLC39A13) is a SLC39A/ZIP family member. It has been documented to participate in various disease-related mechanisms, including Spondylocheirodysplastic-Ehlers-Danlos syndrome (SCD-EDS) [13,14], dermal disorders [15], and hypersensitivity to nutrient deficiency [16]. We previously characterized *Drosophila* ZIP13 (dZIP13, dZip99c), showing that the transporter localizes in the Golgi apparatus, endoplasmic reticulum (ER), and intracellular vesicles, where it transports cytoplasmic iron into the secretory compartment [17]. RNAi-mediated suppression of *dZIP13* expression in the fat body led to significant accumulation of collagen due to the inactivation or iron-dependent lysyl oxidase activity, similar to the phenotypes caused by *ZIP13* mutations in humans [13,17]. Studies have indicated that ZIP13 exhibits aberrant expression in malignancies such as ovarian cancer and fibrosarcoma [18], however, the mechanisms by which ZIP13 influences tumorigenesis remain poorly understood.

The JAK/STAT signalling cascade is a central pathway that responds to an array of cytokines and growth factors, whose abnormal increase can trigger gene transcription [19]. This pathway exhibits context-dependent roles in tumorigenesis, acting either as a tumor suppressor or promoter depending on cellular and microenvironmental conditions [20]. On one hand, the activation of JAK/STAT signalling can enhance immune responses against the tumor under certain conditions [21]; on the other hand, increased levels of the cytokine IL-6 have been implicated as pivotal in the proliferation and maturation of haematological cancers or solid tumors via the JAK/STAT signalling mechanism [22,23]. Mutation of two iron metabolism genes, *ferritin* or *DMT1*, could activate the JAK/STAT pathway and support glioblastoma and colorectal tumor proliferation [24,25], illustrating the importance of iron metabolism in JAK/STAT signalling.

Human cancers often exhibit characteristic DNA and histone methylation changes because of abnormal epigenetic modifications [26]. The human homolog of the *Drosophila* zeste gene enhancer, known as an enhancer of zeste homolog 2 (EZH2), plays key roles in epigenetic regulation [27]. EZH2 is capable of catalyzing the trimethylation of histone 3 at lysine 27, denoted as H3K27me3, an epigenetic modification that represses genes associated with tumor suppression and cellular differentiation [28]. Among the ten-eleven translocation (TET) family of enzymes, TET-1, TET-2, and TET-3 are identified as the principal catalysts for DNA demethylation, which is an essential process in various biological functions, including development, differentiation, and disease processes such as tumor malignancies and invasiveness [27,29,30]. Therefore, investigation of these enzymes’ biological functions and catalytic mechanisms contributes to further understanding of mechanisms of tumor development and can provide a new therapeutic strategy.

*Drosophila melanogaster* exhibits significant conservation towards human genes, molecular mechanisms, functional organs, and biological physiology [31]. Many oncogenes in humans have been found in *Drosophila* [32]. In the present study, we employed a well-established *Drosophila* malignant cancer model in which cells in the eye-antennal imaginal disc co-express oncogenic Raf gain-of-function and loss-of-function mutants in the tumor suppressor Scribbled (Raf^GOF^scrib^−/−^), which was applied in our previous report [33]. The Raf^GOF^scrib^−/−^ model has become a crucial tool for investigating tumor progression mechanisms due to its ability to replicate the growth, invasion, and metastasis characteristics of human tumors [33,34]. Our previous study [33] demonstrated that the knockdown of the zinc transporter *ZnT7* (*ZnT7* RNAi) significantly promotes tumor growth and invasion in the Raf^GOF^scrib^−/−^ model. This effect is mediated through the activation of the JNK signalling pathway—ZnT7 dysfunction, leading to zinc homeostasis imbalance, upregulates JNK pathway activity, thereby enhancing tumor cell proliferation and migration. This finding suggests that metal transporters may play a pivotal role in the malignant progression of Raf^GOF^scrib^−/−^ tumors. The present study further focuses on another metal transporter, dZIP13, to explore its regulatory role and molecular mechanisms in tumor progression within this model, aiming to expand the understanding of the relationship between metal metabolism and tumorigenesis. Our findings show that suppression of *dZIP13* in tumor clones significantly enhances tumor expansion, invasiveness, and metastatic spread. Mechanistically, *dZIP13* RNAi leads to cytosolic iron accumulation, which activates the TET DNA dioxygenase, modulates EZH2 transcription, and ultimately hyperactivates the JAK/STAT signaling pathway to drive tumor progression. This finding thus provides unique insights into understanding how iron participates in tumor progression and suggests an epigenetic regulation role of iron in cancer development.

## Results

### *dZIP13* knockdown promotes tumor growth and invasion

To explore the role of ZIP13 in oncogenesis, we leveraged data from The Cancer Genome Atlas (TCGA) and the cBioPortal to assess the prevalence of ZIP13 mutations. Our findings showed that *ZIP13* mutations are present across a spectrum of cancers, including Uterine Corpus Endometrial Carcinoma, Thymoma, Skin Cutaneous Melanoma, among others. Analysis using the Kaplan-Meier Plotter platform (https://tnmplot.com/analysis/) revealed that SLC39A13 mRNA levels are altered across multiple tumors (S1A Fig). Besides, the expression of SLC39A13 was significantly declined in breast, colon, lung, and ovary tumor tissues (S1A Fig). Collectively, these analyses suggest that SLC39A13 expression is frequently dysregulated in multiple human cancers. 

**Fig 1:**
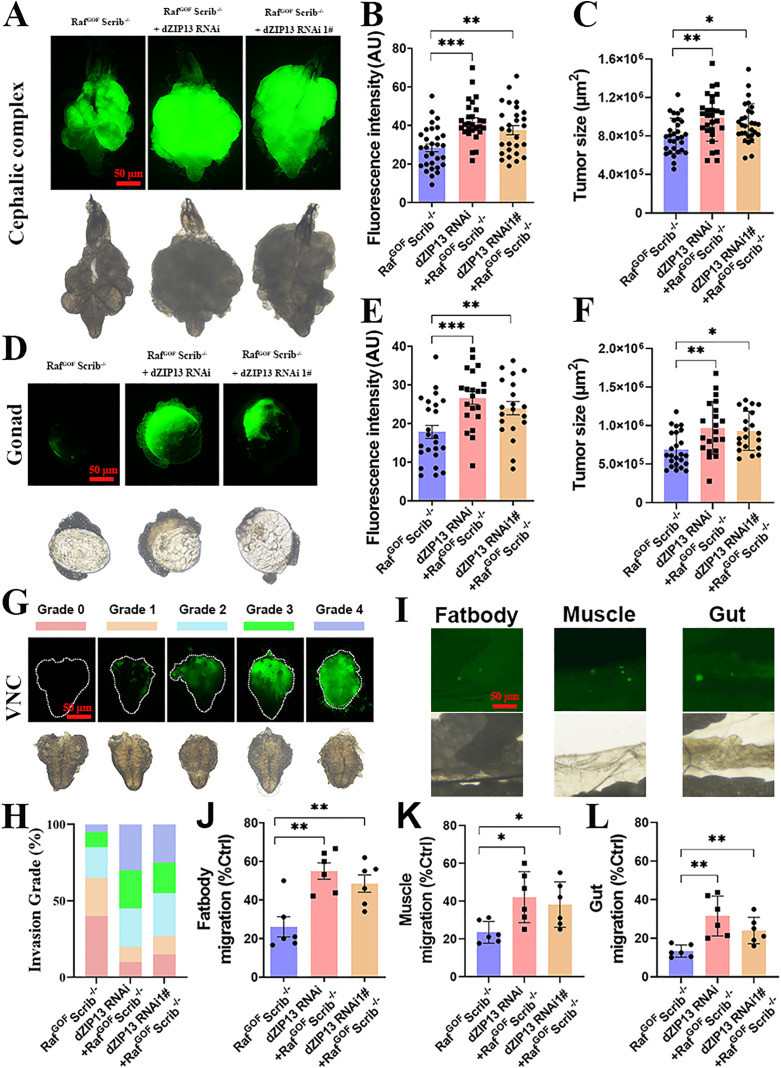
*dZIP13* knockdown promotes RafGOFScrib−/− tumorigenesis and malignant development. **(A)** *dZIP13* RNAi increased tumor overgrowth in the cephalic complex of Raf^GOF^Scrib^−/−^ flies at day 10 after egg laying (AEL). Scale bar, 50 μm. **(B-C)** Relative fluorescence intensity **(B)** and tumor size **(C)** were quantified for the specified genotypes. *n* = 30 (Raf^GOF^Scrib^−/−^ control), *n* = 28 (*dZIP13* RNAi; Raf^GOF^Scrib^−/−^), and *n* = 28 (*dZIP13* RNAi1#; Raf^GOF^Scrib^−/−^). **(D)** Tumor growth in the gonad was exacerbated by *dZIP13* RNAi at 10 days AEL. Scale bar, 50 μm. **(E-F)** Quantification of relative fluorescence intensity **(E)** and tumor size **(F)** for the indicated genotypes. *n* = 24 (Raf^GOF^Scrib^−/−^ control), *n* = 21 (*dZIP13* RNAi; Raf^GOF^Scrib^−/−^), *n* = 20 (*dZIP13* RNAi1#; Raf^GOF^Scrib^−/−^). **(G)** The invasive characteristics of tumors were classified into four grades based on our classification criteria at 10 days AEL. Scale bar, 50 μm. **(H)** Statistical analysis of tumor invasion levels at 10 days AEL. *n* = 32 (Raf^GOF^Scrib^−/−^), *n* =38 (*dZIP13* RNAi; Raf^GOF^Scrib^−/−^), *n* = 36 (*dZIP13* RNAi1#; Raf^GOF^Scrib^−/−^). **(I)** Representative confocal images showing metastatic tumor clones (GFP-positive) in distant tissues of *dZIP13* RNAi; Raf^GOF^Scrib^−/−^ flies. Scale bar, 50 μm. **(J)** Tumor cells invading the fat body. **(K)** Tumor cell migration into the body wall muscle. **(L)** GFP-positive metastatic clones detected in the gut. (%Control indicated that the proportion of invasion into fatbody, muscle and gut in total.) Data are presented as mean ± SEM. Statistical significance was calculated using unpaired two-tailed Student′s *t*-test or chi-square test as appropriate. *p< 0.05, **p< 0.01, ***p< 0.001. Genotypes used are as follows: **(A–L)** *ey-Flp/+; Act>y*^*+*^*-Gal4, UAS-GFP/+*; FRT82B *tub-Gal80/UAS-Raf*^*GOF*^ FRT82B *Scrib*^−/−^, *ey-Flp/+; Act>y*^*+*^*-Gal4, UAS-GFP/dZIP13 RNAi*; FRT82B *tub-Gal80/UAS-Raf*^*GOF*^ *FRT82B Scrib*^−/−^ and *ey-Flp/+; Act>y*^*+*^*-Gal4, UAS-GFP/dZIP13 RNAi1#*; FRT82B *tub-Gal80/UAS-Raf*^*GOF*^ *FRT82B Scrib*^−/−^.

The co-expression of oncogenic Raf gain-of-function (Raf^GOF^) and loss-of-function mutations in the tumor suppressor Scribbled (Scrib^−/−^) in the *Drosophila* eye-antennal imaginal disc (Raf^GOF^Scrib^−/−^) induces aggressive tumor-like overgrowth and systemic dissemination, recapitulating key features of human malignancies [34]. GFP-tagged scrib MARCM clones are produced across various tissues, including the eye imaginal discs, brain neuroepithelium, and gonads [35–37]. These tumors disseminate to additional tissues around day 6 post-oviposition, with the larvae typically perishing around day 15 [35]. Subsequently, we evaluated the impact of *dZIP13* RNAi on Raf^GOF^Scrib^−/−^ at day 10 post-oviposition. As illustrated in Fig 1A-1F, the fluorescence measurements revealed that the suppression of dZIP13 significantly boosted fluorescence intensity in the cephalic complex and gonad (with increases of 48% and 23%, respectively, as shown in Fig 1A, 1B, 1D, and 1E). Additionally, the analysis of tumor dimensions indicated that the volume of tumors in the cephalic complex and gonad was enlarged due to *dZIP13* knockdown (with increases of 49% and 41%, respectively, as depicted in Fig 1A, 1C, 1D, and 1F). Together with the increased tumor volume, these results indicate that *dZIP13* knockdown markedly enhances tumor overgrowth in the Raf^GOF^Scrib^−/−^ background.

The majority of Raf^GOF^Scrib^−/−^ tumors have been observed to spread to the ventral nerve cord (VNC) within the timeframe of days 8–12 post-oviposition [33,38]. The extent of VNC infiltration on day 10 following egg laying serves as a metric for assessing the degree of tumor invasiveness [38]. As shown in Fig 1G, tumor invasion was categorized into four distinct levels: Grade 1 (minimal), Grade 2 (mild), Grade 3 (moderate), and Grade 4 (severe) invasion. As shown in Fig 1H, the suppression of *dZIP13* via RNAi intensifies tumor invasion, with a 20% reduction in the initial stage, a 35% escalation in the mild stage, a 16% augmentation in the moderate stage, and a 51% surge in the severe stage, all in contrast to the Raf^GOF^Scrib^−/−^ model. Moreover, silencing *dZIP13* in the tumor clones promotes more tumors metastasizing to the muscle (increase by ~17.5%), gut (increase by ~20%) and fat-body (increase by ~29.8%) (Fig 1I-1L). In summary, the knockdown of *dZIP13* promotes primary tumor growth and invasion of other tissues. We further performed the overexpression of *dZIP13* in Raf^GOF^Scrib^−/−^ clones using UAS-*dZIP13* lines. Unexpectedly, *dZIP13* overexpression (OE) did not suppress tumor phenotypes, but instead enhanced tumor growth and invasion (S1B-S1D Fig). Together, we observed that both *dZIP13* knockdown and overexpression disrupted normal iron homeostasis and led to phenotypic consequences, suggesting that precise regulation of dZIP13 levels is critical for maintaining iron balance and preventing pathological signalling activation.

### Iron accumulation promotes tumor growth, invasion, and dissemination in *Drosophila*

**Fig 2:**
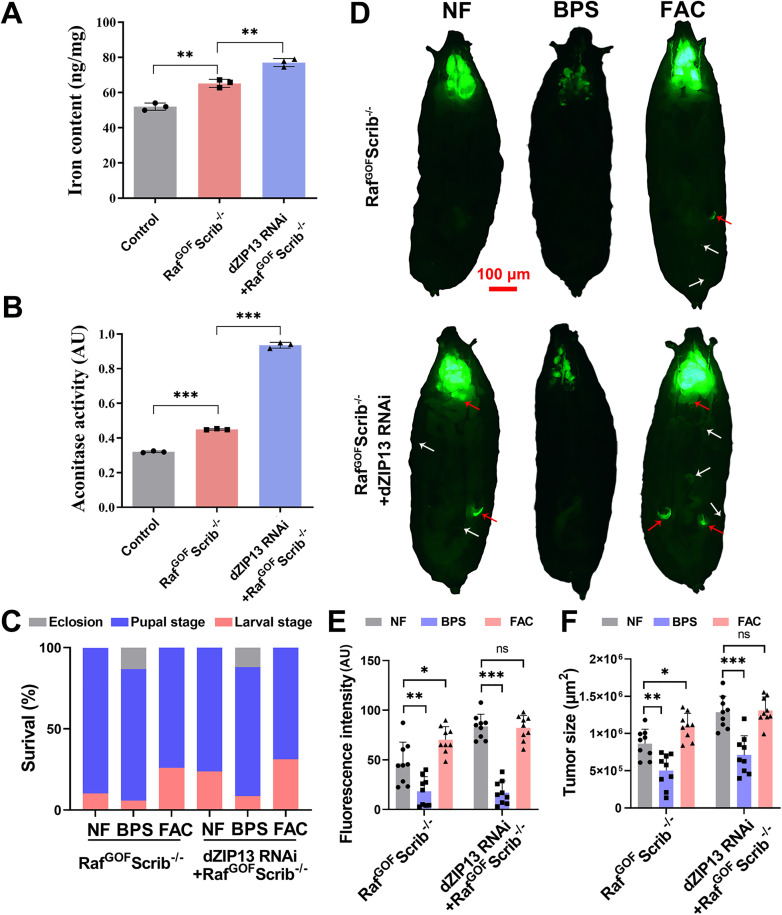
The tumor growth and metastasis in both Raf^GOF^Scrib^−^^/^^−^ and Raf^GOF^Scrib^−^^/^^−^, *dZIP13* RNAi flies (10 d AEL) could be modified by dietary iron. **(A)** Ferrozine assays suggested increased iron content in the cephalic complex of Raf^GOF^Scrib^−/−^, and *dZIP13* RNAi increased the iron content even more. *n* = 150 cephalic complexes per group. **(B)** Aconitase activity demonstrated a marked increase in iron levels within the cephalic complex *dZIP13* RNAi; Raf^GOF^Scrib^−/−^ compared to Raf^GOF^Scrib^−/−^. *n* = 150 cephalic complexes per group. **(C)** The survival rate of Raf^GOF^Scrib^−/−^ and *dZIP13* RNAi; Raf^GOF^Scrib^−/−^ were inhibited by BPS and enhanced by FAC. *n* = 50 larvae per vial, *n* = 6 vials per experimental group. **(D)** Tumor growth (indicated by red arrows) and metastasis (indicated by white arrows) in both Raf^GOF^Scrib^−/−^ and *dZIP13* RNAi; Raf^GOF^Scrib^−/−^ flies (10 d AEL) were effectively inhibited by BPS while significantly enhanced by FAC. Scale bar, 100 μm. "NF" refers to "normal food", which was used as the standard control diet in experiments. **(E-F)** Quantification of relative fluorescence intensity **(E)** and tumor size **(F)** for the specified genotypes. *n* = 9. *p < 0.05, **p < 0.01, ***p < 0.001 and ns no significant. Genotypes used are as follows: **(A–B)** *ey-Flp/ + ; Act > y*^*+*^*-Gal4* (control), *ey-Flp/ + ; Act > y*^*+*^*-Gal4, UAS-GFP/ +* ; FRT82B *tub-Gal80/UAS-Raf*^*GOF*^ FRT82B *Scrib*^−/−^ and *ey-Flp/ + ; Act > y*^*+*^*-Gal4, UAS-GFP/dZIP13 RNAi*; FRT82B *tub-Gal80/UAS-Raf*^*GOF*^ *FRT82B Scrib*^−/−^. **(C-F)** *ey-Flp/ + ; Act > y*^*+*^*-Gal4, UAS-GFP/ +* ; FRT82B *tub-Gal80/UAS-Raf*^*GOF*^ FRT82B *Scrib*^−/−^ and *ey-Flp/ + ; Act > y*^*+*^*-Gal4, UAS-GFP/dZIP13 RNAi*; FRT82B *tub-Gal80/UAS-Raf*^*GOF*^ *FRT82B Scrib*^−/−^.

A growing body of evidence in mammals indicates that iron metabolism plays a crucial role in tumor progression. Our previous work identified dZIP13 as an iron-exporting protein within the secretory pathway [17]. However, it remains unknown whether the regulation of iron metabolism by dZIP13 affects tumor growth and invasion. We therefore quantified intracellular iron content using a ferrozine assay. Fig 2A shows a striking iron accumulation in Raf^GOF^Scrib^−/−^ tumor tissues, in contrast to the control group. Furthermore, iron accumulation was further enhanced in *dZIP13* RNAi; Raf^GOF^Scrib^−/−^ (Fig 2A). We next measured the brain complex aconitase activity to further reflect whether the availability of iron within the cells has changed (Fig 2B). Consistent with the ferrozine, the knocking down of *dZIP13* in Raf^GOF^Scrib^−/−^ results in an approximately 2-fold increase in intracellular iron (Fig 2B).

Approximately 90% Raf^GOF^Scrib^−/−^ animals died at the pupal stage, and ~ 10% died during the larval stage. In contrast, ~70% *dZIP13* RNAi; Raf^GOF^Scrib^−/−^ animals died at the pupal stage, with the remainder dying during the larval stage (Fig 2C). The developmental issues could be somewhat alleviated by incorporating the iron chelator bathophenanthrolinedisulfonic acid disodium (0.1 mM BPS) into the diet, whereas they were exacerbated by the dietary supplementation of ferric ammonium citrate (5 mM FAC) (Fig 2D). Subsequently, we assessed the impact of dietary iron manipulation on tumorigenesis and invasiveness. Tumor growth and invasion in both Raf^GOF^Scrib^−/−^ and *dZIP13* RNAi; Raf^GOF^Scrib^−/−^ larvae were significantly attenuated by BPS and exacerbated by FAC (Fig 2C-2F). Increasing iron content by genetic modulation of ZIP13 or dietary iron intervention with iron supplementation could exacerbate tumor growth and migration [17,39]. The dietary intervention of iron uptake with iron chelators in Raf^GOF^Scrib^−/−^ or *dZIP13* RNAi; Raf^GOF^Scrib^−/−^ alleviated the tumor progression. These data further strengthen the notion that iron is involved in the growth and invasion of tumors *in vivo*. These data support the notion that iron accumulation caused by *dZIP13* RNAi contributes to malignant tumor development in the Raf^GOF^Scrib^−/−^ background.

### Cytosolic iron accumulation caused by *dZIP13* RNAi promotes tumor growth and invasion via the JAK/STAT signalling pathway

Several iron metabolism genes, including hepcidin, transferrin receptor one and ferritin, were reported to stimulate the JAK/STAT, IL6/JAK2/STAT3 signalling pathway [24], and JAK/STAT signalling is closely related to tumor development [22]. We therefore hypothesized that *dZIP13* RNAi may promote tumorigenesis via the JAK/STAT pathway. Consequently, we utilized the STAT92E-GFP reporter [40] to investigate the influence of *dZIP13* RNAi on JAK/STAT signalling in the eye discs of *Drosophila* larvae (S2A-S2B Fig). Our findings indicated that *dZIP13* RNAi triggers JAK/STAT pathway activation, and the overactivation of this pathway due to *dZIP13* RNAi could be mitigated by BPS and intensified by FAC (S2A-S2B Fig).

**Fig 3:**
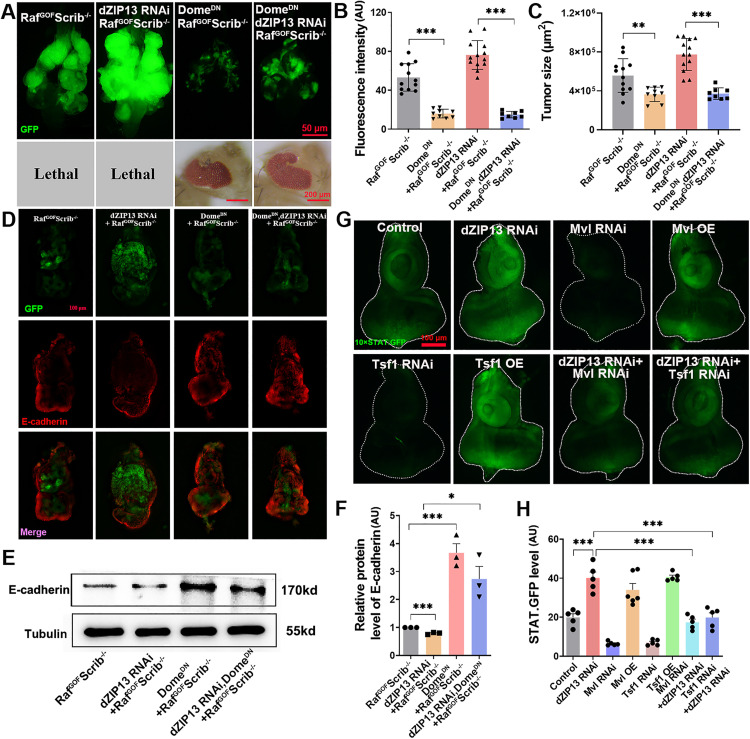
*dZIP13* knockdown enhances tumor growth and invasion through JAK/STAT signalling pathway activation, which results from cytosolic iron accumulation. **(A)** The tumor growth and invasion induced by *dZIP13* RNAi were notably inhibited by disrupting the JAK/STAT signalling pathway through the co-expression of Dome^DN^. Scale bar, 50 μm. **(B-C)** Relative fluorescence intensity **(B)** and tumor size **(C)** were quantified for the specified genotypes. *n* = 12 (Raf^GOF^Scrib^−/−^ control), *n* = 8 (Dome^DN^; Raf^GOF^Scrib^−/−^), *n* = 13 (*dZIP13* RNAi; Raf^GOF^Scrib^−/−^), *n* = 9 (Dome^DN^, *dZIP13* RNAi; Raf^GOF^Scrib^−/−^). **(D)** Immunohistochemical staining demonstrates that *dZIP13* RNAi leads to a decrease in E-cadherin expression, which can be rescued by Dome^DN^. Scale bar, 200 μm. **(E)** Western blot analysis demonstrates that dZIP13 RNAi leads to a decrease in E-cadherin expression, which can be rescued by Dome^DN^. **(F)** Quantification the E-cadherin level of the indicated genotypes. **(G)** Compared with the control, *dZIP13* RNAi, *Mvl* OE and *Tsf1* OE induced 10 × STAT.GFP expression in the eye-antennal discs. *Mvl* RNAi or *Tsf1* RNAi suppressed the induced 10 × STAT.GFP in *dZIP13* RNAi. Scale bar, 100 μm. **(H)** Quantification of STAT.GFP intensity for the specified genotypes. *p < 0.05, **p < 0.01, ***p < 0.001. Genotypes used are as follows: **(A-F)** *ey-Flp/ + ; Act > y*^*+*^*-Gal4, UAS-GFP/ +* ; FRT82B *tub-Gal80/UAS-Raf*^*GOF*^ FRT82B *Scrib*^−/−^, *ey-Flp/ + ; Act > y*^*+*^*-Gal4, UAS-GFP/dZIP13 RNAi*; FRT82B *tub-Gal80/UAS-Raf*^*GOF*^ *FRT82B Scrib*^−/−^, *ey-Flp/ + ; Act > y*^*+*^*-Gal4, UAS-GFP/UAS-Dome*^*DN*^; FRT82B *tub-Gal80/UAS-Raf*^*GOF*^ FRT82B *Scrib*^−/−^ and *ey-Flp/ + ; Act > y*^*+*^*-Gal4, UAS-GFP/UAS-Dome*^*DN*^*,dZIP13 RNAi*; FRT82B *tub-Gal80/UAS-Raf*^*GOF*^ *FRT82B Scrib*^−/−^. **(G-H)** *ey-gal4/ + ; 10 × STAT.GFP/+* (control), *ey-gal4/dZIP13 RNAi*; *10 × STAT.GFP/ +* , *ey-gal4/Mvl OE; 10 × STAT.GFP/ +* , *ey-gal4/UAS-Tsf1 OE*; *10 × STAT.GFP/ +* , *ey-gal4/dZIP13 RNAi; 10 × STAT.GFP/Mvl RNAi* and *ey-gal4/dZIP13 RNAi; 10 × STAT.GFP/Tsf1-RNAi*.

Domeless (dome) encodes the only transmembrane receptor of the JAK/STAT pathway in *Drosophila*. To determine whether JAK/STAT signalling is functionally required for the tumor-promoting effects of *dZIP13* RNAi, we expressed a dominant-negative form of the JAK/STAT receptor Domeless (Dome^DN^). The results demonstrated that the tumor growth and invasion (Fig 3A-3C), as well as the survival (S2C Fig) of both Raf^GOF^Scrib^−/−^ and *dZIP13* RNAi; Raf^GOF^Scrib^−/−^ were markedly inhibited by Dome^DN^. These findings indicate that the JAK/STAT signalling contributes to the tumor-promoting effects of *dZIP13* RNAi on tumor growth and migration.

The initiation of the epithelial-mesenchymal transition (EMT) process is pivotal for the spread of cancer cells. Loss or reduction of E-cadherin expression is commonly used as a hallmark of EMT initiation [41]. High-magnification confocal images of eye–antennal discs were used to visualize E-cadherin expression at the cellular level (Fig 3D). In line with the intensified tumor growth and migration, *dZIP13* RNAi led to a decrease in E-cadherin expression within tumor clones (Fig 3D-3F). Moreover, the reduced E-cadherin expression in *dZIP13* RNAi; Raf^GOF^Scrib^−/−^ was rescued by Dome^DN^, indicating that the persistent stimulation of the JAK/STAT pathway due to dZIP13 RNAi results in the suppression of E-cadherin expression, thereby promoting excessive tumor proliferation and invasion.

As previously stated, dZIP13 functions as an iron importer into the secretory pathway, transporting Fe from the cytoplasm into secretory organelles such as the ER/Golgi. Therefore, knockdown of *dZIP13* leads to a reduction of iron within the secretory pathway and a concomitant accumulation of iron in the cytoplasm. The *Drosophila* eye disc has proven to be a potent model for investigating the JAK/STAT signalling pathway [42]. The STAT.GFP reporter allows for the detection of JAK/STAT pathway activation in *Drosophila* [34]. To delve deeper into whether the activation of the JAK/STAT pathway by *dZIP13* RNAi is associated with iron deficiency in the secretory pathway or iron accumulation in the cytoplasm, we altered the expression of many iron transporters, specifically in the eye progenitor cells by crossing these transgenic flies to Eyeless-Gal4 [43]. *Drosophila* Malvolio (Mvl) encodes an SLC11 family of metal ion transporters member that transports divalent metal cations in cells, including iron [44]. Transferrin 1 (Tsf1) transports iron between tissues like mammalian serum transferrin [39]. The expression of STAT.GFP was upregulated in *dZIP13* RNAi, *Malvolio* overexpression (*Mvl* OE) or *Transferrin 1* overexpression (*Tsf1* OE), compared with the control (Fig 3G-3H). Besides, the increased STAT.GFP fluorescence in *dZIP13* RNAi could be restored by *Mvl* RNAi or *Tsf1* RNAi (Fig 3G-3H). Aconitase activity positively correlates to the cytosolic iron level [45]. As shown in S2D Fig, compared to the control, aconitase activity was induced by *dZIP13* RNAi, *Mvl* OE or *Tsf1* OE (~23%, ~ 30% and ~29%, respectively). The increased aconitase activity in *dZIP13* RNAi was consistently restored by *Mvl* RNAi or *Tsf1* RNAi (S2D Fig). These data suggest that the increased cytosolic iron levels contribute to JAK/STAT signalling activation.

### EZH2 mediates the JAK/STAT signalling activation caused by *dZIP13* RNAi

**Fig 4:**
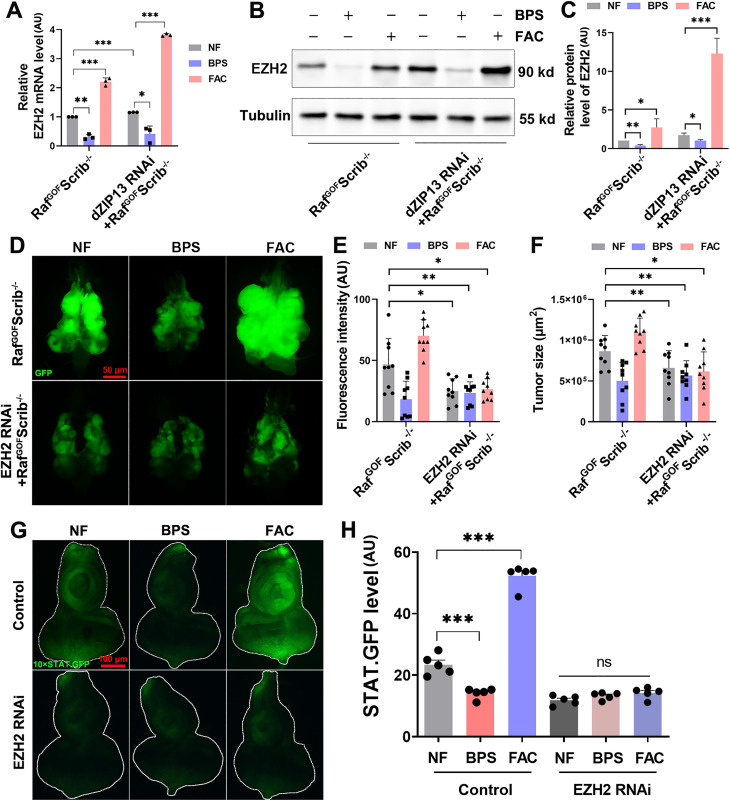
The activation of iron on JAK/STAT signalling and tumor progression depends on EZH2. **(A)** qPCR showed *dZIP13* RNAi and the addition of extra FAC increased the EZH2 mRNA level, whereas BPS dramatically down-regulated it in Raf^GOF^Scrib^−/−^ tumors. *n* = 50 cephalic complexes per group. **(B)** Western blot analysis reveals that the dietary iron chelator BPS significantly reduced the levels of EZH2 in tumors, while *dZIP13* RNAi and the addition of extra FAC led to an increase in these levels. *n* = 50 cephalic complexes per group. **(C)** Quantitative assessment of EZH2 levels from **(B)**. **(D)** The effect of iron on tumor growth and invasion was lost following the knockdown of *EZH2*. Scale bar, 50 μm. **(E-F)** Quantification of relative fluorescence intensity **(E)** and tumor size **(F)** for the specified genotypes. *n* = 9 per group. **(G)** The influence of iron on 10 × STAT.GFP expression was absent in *EZH2* RNAi. Scale bar, 100 μm. (H) Quantification of STAT.GFP intensity for the indicated genotypes. *n* = 6. *p < 0.05, **p < 0.01 and ***p < 0.001. Genotypes used are as follows: **(A-C)** *ey-Flp/ + ; Act > y*^*+*^*-Gal4, UAS-GFP/ +* ; FRT82B *tub-Gal80/UAS-Raf*^*GOF*^ FRT82B *Scrib*^−/−^*.* **(D-F)** *ey-Flp/ + ; Act > y*^*+*^*-Gal4, UAS-GFP/ +* ; FRT82B *tub-Gal80/UAS-Raf*^*GOF*^ FRT82B *Scrib*^−/−^ and *ey-Flp/ + ; Act > y*^*+*^*-Gal4, UAS-GFP/EZH2 RNAi*; FRT82B *tub-Gal80/UAS-Raf*^*GOF*^ *FRT82B Scrib*^−/−^. **(G-H)** *ey-gal4/ + ; 10 × STAT.GFP/+* (control) and *ey-gal4/EZH2 RNAi*; *10 × STAT.GFP/ +* .

The experiments showed that cytosolic iron accumulation is sufficient to promote tumor progression by activating JAK/STAT signalling. However, how iron activates JAK/STAT signalling remains unclear. Epigenetics modifications are closely associated with tumorigenesis, invasion and metastasis [46]. In a related, ongoing study, we screened for chromatin regulators whose expression is sensitive to iron levels. Our screening found that the expression of *Drosophila* EZH2 could be modulated by iron in normal conditions (S3A-S3B Fig). Moreover, iron can bind to various proteins to affect their structure or activity [1]. To further identify the mechanism underlining how iron regulates EZH2 expression, we analyzed the 3D structure of EZH2 protein (UniProt: P42124) in AlphaFold Protein Structure Database (S4A Fig). We next searched the iron binding site of the EZH2 protein via SWISS-MODEL (https://swissmodel.expasy.org/). However, no iron binding site was found in EZH2. It suggests that iron may indirectly affect EZH2 protein levels. We therefore tested whether EZH2 is regulated at the transcriptional and/or post-transcriptional level by iron. As shown in Fig 4A, EZH2 mRNA levels could be induced by FAC and inhibited by BPS in Raf^GOF^Scrib^−/−^. Moreover, the protein expression of EZH2 could be inhibited by BPS and enhanced by FAC (Fig 4B-4C). Together, these data indicate that iron positively regulates EZH2 expression at both the mRNA and protein levels in this context.

We subsequently examined the effects of EZH2 on iron-induced tumor progression. As mentioned before, iron reduction significantly rescued, whereas iron accumulation exacerbated the tumor growth and invasion (Fig 4D-4F). As shown in Fig 4D-4F, *EZH2* RNAi significantly inhibited tumor growth and invasion in Raf^GOF^Scrib^−/−^. Remarkably, when *EZH2* was knocked down, altered iron levels in tumors no longer significantly affected tumor progression, including the tumor growth, invasion and metastasis (Figs 4D-4F and S4B-S4C), suggesting that EZH2 is required for iron-mediated regulation of tumor progression *in vivo*.

To explore if the regulation of iron on JAK/STAT depends on EZH2, we examined the impact of *EZH2* RNAi on the levels of 10 × STAT.GFP in the eye discs (Fig 4G-4H). Consistent with the results described above, both STAT.GFP (Fig 4G-4H) and expression of the STAT target gene chinmo (S4D Fig) indicate that the effect of iron on the JAK/STAT pathway was abolished upon *EZH2* knockdown. Collectively, these findings demonstrate that the iron-induced activation of the JAK/STAT pathway in tumors is EZH2-dependent.

### *dZIP13* knockdown promotes EZH2 expression by increasing TET enzyme activity

**Fig 5:**
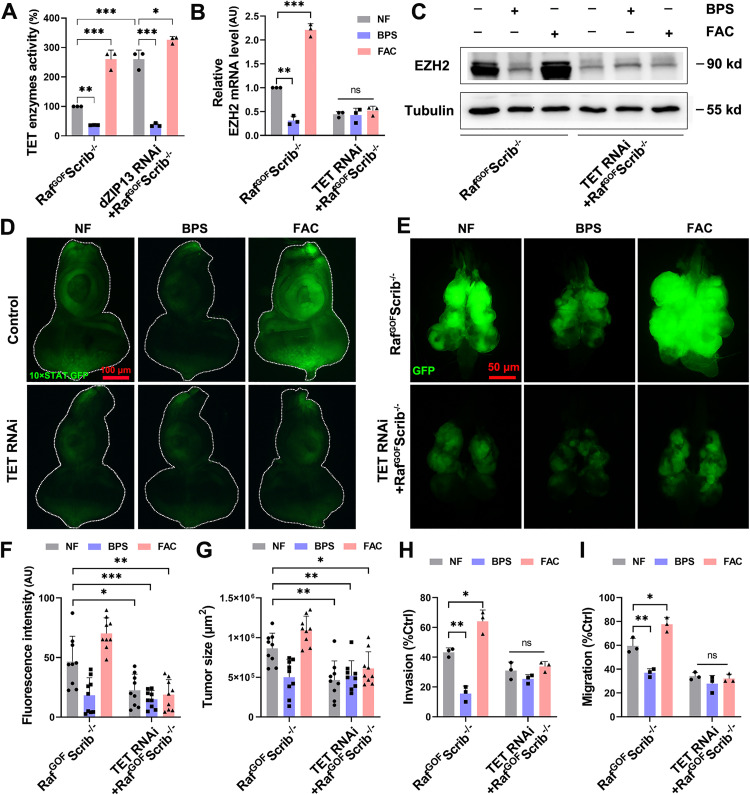
Iron regulates the transcription of EZH2 through TET. **(A)** The TET enzyme activity was considerably diminished in Raf^GOF^Scrib^−/−^ fed with BPS while increased under *d*ZIP*13* RNAi or fed with FAC in Raf^GOF^Scrib^−/−^. *n* = 50 cephalic complexes per group. **(B)** The mRNA levels of *EZH2* in tumors were significantly decreased by BPS and markedly increased by FAC. UAS-*TET* RNAi suppressed the regulation of iron on *EZH2* expression. *n* = 150 cephalic complexes per group. **(C)** Dietary iron supplementation elevated the expression of EZH2 in the tumors, whereas iron chelator BPS reduced its expression. EZH2 level was reduced in UAS-*TET* RNAi, and UAS-*TET* RNAi inhibited the regulation of iron on EZH2 expression. **(D-E)** Both the activated JAK/STAT signalling **(D)** and tumor progression **(E)** were suppressed by UAS-*TET* RNAi, and the regulation of iron on JAK/STAT signalling and tumor progression disappeared in UAS-*TET* RNAi. Scale bar, 100 μm. **(F-G)** Quantification of relative fluorescence intensity **(F)** and tumor size **(G)** for the specified genotypes. *n* = 9 per group. **(H-I)** The regulation of iron on tumor invasion **(H)** and migration **(I)** disappeared in UAS-*TET* RNAi. *n* = 30 animals were used, derived from three separate experiments. *p < 0.05, **p < 0.01, ***p < 0.001 and ns no significant. Genotypes were as follows: **(A)** *ey-Flp/ + ; Act > y*^*+*^*-Gal4, UAS-GFP/ +* ; FRT82B *tub-Gal80/UAS-Raf*^*GOF*^ FRT82B *Scrib*^−/−^. **(B-C)** *ey-Flp/ + ; Act > y*^*+*^*-Gal4, UAS-GFP/ +* ; FRT82B *tub-Gal80/UAS-Raf*^*GOF*^ FRT82B *Scrib*^−/−^ and *ey-Flp/ + ; Act > y*^*+*^*-Gal4, UAS-GFP/*UAS-*TET* RNAi; FRT82B *tub-Gal80/UAS-Raf*^*GOF*^ *FRT82B Scrib*^−/−^. **(D)** *ey-gal4/ + ; 10 × STAT.GFP/+* (control) and *ey-gal4/*UAS-*TET* RNAi; *10 × STAT.GFP/ +* . **(E-I)** *ey-Flp/ + ; Act > y*^*+*^*-Gal4, UAS-GFP/ +* ; FRT82B *tub-Gal80/UAS-Raf*^*GOF*^ FRT82B *Scrib*^−/−^ and *ey-Flp/ + ; Act > y*^*+*^*-Gal4, UAS-GFP/*UAS-*TET* RNAi; FRT82B *tub-Gal80/UAS-Raf*^*GOF*^ *FRT82B Scrib*^−/−^.

Human ten-eleven translocation (TET) enzymes are a family of Fe(II)/α-ketoglutarate–dependent dioxygenases that catalyze DNA [29,47]. *Drosophila* encodes a single TET homolog (CG43444). We further found that the regulation of iron on EZH2 expression depends on TET in *Drosophila* tumor tissues. We found *Drosophila* TET enzyme activity could be induced by FAC and inhibited by BPS in Raf^GOF^Scrib^−/−^ (Fig 5A). We next investigated if iron-dependent TET activity is responsible for the increased EZH2 level and activated JAK/STAT signalling. As shown in Fig 5B-5C, the mRNA and protein levels of EZH2 were lowered in *TET* RNAi, and the enhancement of EZH2 expression due to iron enrichment was eliminated following *TET* knockdown in Raf^GOF^Scrib^−/−^ tumors. Consistently, JAK/STAT activation induced by iron was also inhibited by *TET* RNAi (Fig 5D). Remarkably, the tumor growth, invasion and dissemination were markedly suppressed by *TET* RNAi in Raf^GOF^Scrib^−/−^ larvae (Fig 5E-5G). Moreover, iron depletion (BPS) or supplementation (FAC) no longer affects tumorigenesis and progression when *TET* is knocked down (Fig 5H-5I). These results provide direct evidence that TET is required for iron-regulated EZH2 expression, which is accountable for the activation of JAK/STAT signalling in tumors.

### The hemocyte recruitment and proliferation caused by *dZIP13* RNAi promote oncogenic growth

The JAK/STAT signalling in *Drosophila* is triggered by the unpaired (upd) gene family, which includes upd1, upd2, and upd3, and is analogous to human interleukin 6 [34]. 

Prior studies have shown that an affirmative feedback loop, characterized by the activation of the JAK/STAT pathway, operates between neoplastic cells and blood cells, thereby fostering the proliferation of tumors: Overactivation of JAK/STAT in cancerous tissues stimulates the secretion of upd cytokines, subsequently activating JAK/STAT signalling in the hemocytes [23]; the BM disruption in tumor tissues and JAK/STAT activation in the hemocyte subsequently promotes hemocyte recruitment and proliferation [23]. Upon recruitment, hemocytes contribute to tumor advancement by releasing soluble signalling molecules [23].

**Fig 6:**
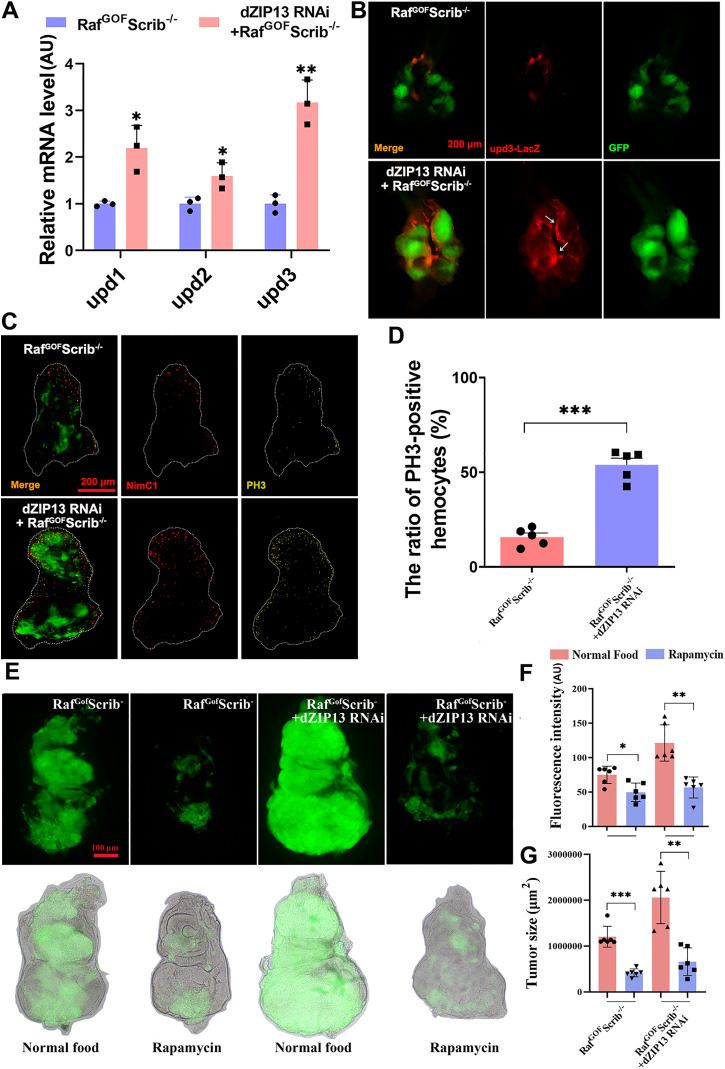
The exacerbation of tumor malignant development by *dZIP13* RNAi involves the recruitment and proliferation of hemocytes, which are mediated by upds. **(A)** The upds mRNA levels were increased in *dZIP13* RNAi tumors. *n* = 50 cephalic complexes per group. **(B)** The upd3-lacZ reporter expression revealed an increase in upd3 protein levels within *dZIP13* RNAi-associated tumors. Scale bar, 200 μm. **(C)** Co-immunostaining showing an increased number of hemocytes (red, anti-NimC1) adhering to the surface of eye-antennal discs in Raf^GOF^Scrib^−/−^ and *dZIP13* RNAi;Raf^GOF^Scrib^−/−^ tumors. Scale bar: 200 μm. **(D)** The ratio of PH3-positive hemocytes to the total hemocyte population was quantified in Raf^GOF^Scrib^−/−^ and *dZIP13* RNAi; Raf^GOF^Scrib^−/−^ eye-disc tumor clones.stained with an anti-PH3 antibody in eye disc. Scale bar, 200 μm. **(E)** Eye-antennal discs bearing *dZIP13* RNAi; Raf^GOF^Scrib^−/−^ clones were treated with 1% DMSO (control) or rapamycin. **(F-G)** Tumor size and invasive behavior were assessed by GFP labeling of clones. Compared to control, rapamycin treatment markedly reduced clone overgrowth and invasion into surrounding tissues. Quantification of tumor size **(F)** and invasion frequency **(G)** following treatment. *n* = 6. *p < 0.05, **p < 0.01, ***p < 0.001 and ns no significant. Genotypes used are as follows: **(A-C)** *ey-Flp/ + ; Act > y*^*+*^*-Gal4, UAS-GFP/ +* ; FRT82B *tub-Gal80/UAS-Raf*^*GOF*^ FRT82B *Scrib*^−/−^ and *ey-Flp/ + ; Act > y*^*+*^*-Gal4, UAS-GFP/EZH2 RNAi*; FRT82B *tub-Gal80/UAS-Raf*^*GOF*^ *FRT82B Scrib*^−/−^. **(D-G)** *ey-Flp/ + ; Act > y*^*+*^*-Gal4, UAS-GFP/ +* ; FRT82B *tub-Gal80/UAS-Raf*^*GOF*^ FRT82B *Scrib*^−/−^ and *ey-Flp/ + ; Act > y*^*+*^*-Gal4, UAS-GFP/dZIP13 RNAi*; FRT82B *tub-Gal80/UAS-Raf*^*GOF*^ *FRT82B Scrib*^−/−^.

Our studies revealed that the JAK/STAT pathway activation due to iron buildup in *dZIP13* RNAi fosters tumor growth. This finding led us to explore the impact of *dZIP13* RNAi on upds production. Consistent with the influence of *dZIP13* RNAi on JAK/STAT signalling (as seen in Fig 3F), quantitative PCR (qPCR) analysis indicated an upregulation in the mRNA levels of upds (upd1, upd2, and upd3) in *dZIP13* RNAi conditions, with increases of approximately 2.2-, 1.6-, and 3.2-fold, respectively, compared with Raf^GOF^Scrib^−/−^ controls (Fig 6A). An upd3-lacZ transcription reporter [48] was used to confirm this result further. More upd3-lacZ positive cells were identified at the boundary between tumor clones (white arrow) and neighbouring cells (red arrow) in *dZIP13* RNAi; Raf^GOF^Scrib^−/−^, suggesting the upregulation of upd3 in *dZIP13* RNAi (Fig 6B). These data suggested more upds production in *dZIP13* RNAi; Raf^GOF^Scrib^−/−^ tumors. To investigate whether knockdown of *upd3* suppresses tumor growth in the *dZIP13* RNAi; Raf^GOF^Scrib^−/−^ background, we generated flies co-expressing *upd3* RNAi in the same clones (S5A-S5C Fig). The results showed that simultaneous knockdown of *upd3* significantly reduced tumor overgrowth and invasion compared to controls. These findings support the idea that upd3-mediated JAK/STAT activation is a key driver of tumor progression in this model.

We next investigated whether the tumor-promoting effect of *dZIP13* knockdown involves hemocytes. To visualize these cells, we used the anti-NimC1 antibody [49], a well-established marker for circulating and tumor-associated hemocytes. Co-immunostaining with anti-NimC1 and anti-PH3 confirmed that the proliferating cells adhering to the tumor surface were hemocytes (Fig 6C-6D). A markedly increased number of NimC1-positive hemocytes was observed surrounding the *dZIP13* RNAi; Raf^GOF^Scrib^−/−^ tumors compared with controls, indicating enhanced hemocyte recruitment and proliferation at malignant sites.

The above results support activation of the JAK/STAT signalling pathway between tumor cells and hemocytes in the *dZIP13* RNAi; Raf^GOF^Scrib^−/−^ context, and indicate that *dZIP13* RNAi promotes hemocyte recruitment and proliferation. To further clarify the role of hemocytes in tumor growth and invasion, we treated flies with rapamycin, an immunosuppressant known to reduce hemocyte [50]. The results showed that rapamycin treatment significantly suppressed tumor growth and invasion in the *dZIP13* RNAi; Raf^GOF^Scrib^−/−^ background (Fig 6E-6G). Rapamycin treatment significantly reduced the percentage of PH3-positive hemocytes relative to the total hemocyte population adhering to tumor surfaces (S6 Fig). These results suggest that the inhibition of tumor progression by rapamycin is at least partly due to the suppression of hemocyte proliferation at malignant sites.

Finally, we investigate whether the effects of iron on upd expression are mediated by EZH2. As shown in S7A-S7C Fig, the expression of all upds was induced by FAC while reduced by BPS. Furthermore, the regulation of dietary iron intervention on upds completely disappeared when *EZH2* was knocked down in Raf^GOF^Scrib^−/−^ tumors (S7A-S7C Fig). These findings provide additional evidence that the JAK/STAT activation feedback loop between tumor cells and hemocytes is modulated by iron levels, with EZH2 acting as a key mediator of this interaction.

## Discussion

**Fig 7:**
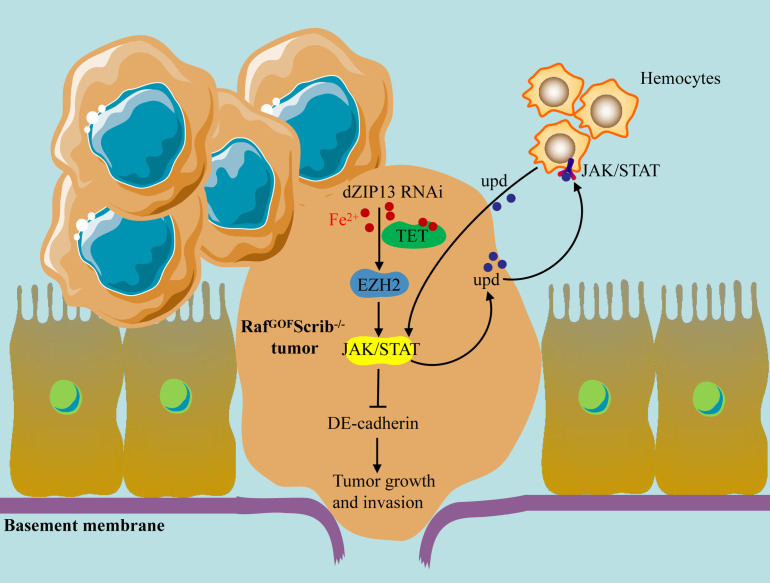
A schematic representation of the mechanisms by which the iron transporter dZIP13 influences tumor development. Iron is an essential regulator of TET activity *in vivo*. The knockdown of *dZIP13* causes iron to accumulate in the cytosol, leading to an increase in TET activity. TET subsequently induces *EZH2* transcription. EZH2 regulates JAK/STAT activation in tumors, resulting in hemocyte recruitment.

Iron is essential for all organisms. Nevertheless, excessive dietary iron intake or an excess of iron in the body correlates with an increased likelihood of developing multiple illnesses, encompassing both the initiation and progression of cancers [51]. Precise nutrition strategies aimed at normalizing intracellular iron levels have not been widely used because the detailed mechanism underlying the regulation of iron on tumor genesis and progression still needs further research. Drawing from *Drosophila* tumor models, this research offers compelling evidence that dZIP13 knockdown increased tumor growth and invasiveness in the Raf^GOF^Scrib^−/−^ model. Further study showed that *dZIP13* knockdown results in cytoplasmic iron accumulation, triggering ten-eleven translocation (TET) activation and enhancer of zeste homolog 2 (EZH2) transcription. Elevated EZH2 levels then promoted JAK/STAT signalling activation through epigenetic modifications. A positive feedback loop in JAK/STAT signalling mediated by upd cytokines further promotes malignant tumor progression (Fig 7). While knockdown of *dZIP13* leads to cytosolic iron accumulation and tumor promotion, overexpression of dZIP13 unexpectedly also exacerbated tumor growth. This suggests that both insufficient and excessive dZIP13 activity may disrupt intracellular iron homeostasis. As dZIP13 mediates the transport of cytosolic iron into the secretory pathway, its overexpression could cause excessive depletion of cytosolic iron or iron overload in secretory compartments. Either condition may disturb cellular redox balance or activate stress-related signalling pathways, thereby promoting tumor progression. These findings underscore the importance of maintaining proper dZIP13 expression levels for iron homeostasis and tumor control. This bidirectional effect of dZIP13 expression may help explain the heterogeneous ZIP13 expression patterns observed in human cancers [18,52]. Thus, rather than functioning as a classical tumor suppressor, dZIP13 may act as a gatekeeper of iron compartmentalization, whose dysregulation in either direction can promote tumorigenesis.

While ZnT7 primarily influences tumor progression via JNK-mediated regulation of proliferation and migration-related genes [33], neither study addresses epigenetic regulation or immune cell involvement. This highlights that metal metabolism is a critical microenvironmental determinant of tumor progression in the Raf^GOF^Scrib^−/−^ model. The differences between the two studies underscore the diversity in how various metal ions regulate tumor progression through distinct pathways, while their commonality emphasizes the metal metabolism network as a core regulatory system in cancer. Zinc and iron, as essential cofactors, disrupt homeostasis through JNK and JAK/STAT pathways, respectively, promoting tumorigenesis. This suggests that tumor cells may adapt to microenvironmental stresses by selectively utilizing specific metal ions to activate distinct signalling pathways at different stages or in specific microenvironments. Although ZnT7 and dZIP13 target different metals, both initiate downstream signalling through abnormal intracellular metal distribution, emphasizing that the subcellular localization of transporters (e.g., secretory pathways) is key to determining metal-specific functions. Alterations in metal distribution, either through loss in secretory pathways or accumulation in the cytoplasm, can selectively activate signalling pathways by altering enzyme activity or protein stability. Both studies suggest that targeting the metal metabolism network — rather than focusing on individual metals or transporters — could be a more effective strategy for tumor therapy. For instance, co-regulating zinc and iron transporters, or simultaneously inhibiting JNK and JAK/STAT pathways, may block the tumor′s ‶metal adaptation″ mechanisms.

Suppressing the JAK/STAT signalling pathway presents an appealing treatment approach for a variety of illnesses. JAK/STAT signalling can induce many genes crucial for controlling cell proliferation, increasing tumor cell migration, and promoting immune infiltration [22,53]. JAK inhibitors are extensively employed in the treatment of certain medical conditions [54,55]. However, there are still many problems with using JAK inhibitors to treat cancer [56]. In this study, we demonstrated that iron accumulation due to *ZIP13* RNAi promotes tumor progression in Raf^GOF^Scrib^−/−^ cases via the JAK/STAT signalling pathway, a process that EZH2 mediates. However, the epigenetics modification of STAT mediated by EZH2 remains unclear. Our study does not specify how EZH2 regulates the JAK/STAT pathway. Other factors involved in this process and the underlying mechanisms need further clarification.

Inhibition of EZH2 has been shown to lead to the regression of many human tumors [57]. Therefore, EZH2 has become a new therapeutic target for tumors [58]. Due to the poor clinical efficacy of EZH2 inhibitors, existing treatments have focused on combination therapy or finding effective new inhibitors. While numerous investigations have centred on how EZH2 modulates translation, there is a scant understanding regarding the regulation of EZH2 itself. Here, we reported that intracellular iron levels could regulate EZH2 expression. To our knowledge, this is the first report of metal regulation on EZH2 to be known. Metal ions are cofactors for many enzymes involved in many biological processes [1]. Here, we showed that TET mediates the regulation of iron on EZH2. We cannot exclude the possibility of other proteins mediating this process. The mechanisms by which TET regulates EZH2 expression remain unclear.

The findings in this study suggest that intracellular iron accumulation contributes significantly to tumor pathology. Iron is a major contributor to free radical generation in many biochemical systems. Excessive iron ions cause oxidative stress reactions, damaging cells (such as endothelial cells, liver cells, pancreatic islet cells, etc.) and promoting cell death and tissue damage [3]. Accumulating evidence suggests that excessive iron is closely associated with various diseases, such as neurodegenerative diseases, cardiovascular and cerebrovascular diseases [59–62]. Besides, iron catalyzes a wide range of biosynthetic processes, including collagen synthesis (lysyl hydroxylases and prolyl hydroxylases), DNA hydroxylation (ten–eleven translocation proteins), histone demethylation (Jumonji proteins), and fatty acid metabolism (trimethyl lysine dioxygenase) [1]. Whether these diverse disorders share common pathogenic mechanisms related to iron-dependent epigenetic regulation warrants further investigation.

In mammals, IL-6 interacts with its receptors, thereby activating the extracellular-signal-regulated kinase/mitogen-activated protein kinase (ERK/MAPK) pathway, as well as the JAK/STAT signalling pathway [63,64]. We further revealed that iron accumulation in tumors activates hemocyte recruitment and proliferation. This process involves excessive secretion of upd cytokines from malignant tumors driven by a JAK/STAT feedforward loop [23,65]. This indicates the regulation of iron on tumor interactions with microenvironmental hemocytes. Intriguingly, either blocking JAK signalling or eliminating the upd level of the tumor leads to a notable increase in cancer burden in the animal. The upd3 is upregulated specifically at the boundaries between clones and neighboring cells as previously reported [66]. This boundary-specific upregulation may reflect intercellular signalling or stress responses triggered by cell competition or altered cell-cell interactions at the clone interfaces [67,68]. Although we cannot exclude partial TOR inhibition in tumor cells, the concomitant reduction of hemocytes and PH3-positive cells supports an immune-suppressive contribution.

To conclude, genetic epistasis analyses position dZIP13 upstream of EZH2, acting as a regulator of its expression via iron–TET signalling. The results of this research suggest that the buildup of iron in the cytoplasm fosters the advancement of tumors through the activation of the JAK/STAT signalling feedforward loop between cancer cells and hemocytes (Fig 7). The regulation of iron on JAK/STAT signalling involves EZH2. The DNA demethylase TET, which needs iron as a cofactor, is responsible for the transcription regulation of EZH2. Considering the conserved mechanisms of epigenetic regulation and JAK/STAT signalling pathways between *Drosophila* and other vertebrates, it is likely that analogous iron regulation mechanisms are present in humans. Furthermore, our results indicate that *Drosophila* serves as a robust model for enhancing our understanding of human cancer biology.

## Materials and methods

### *Drosophila* strains

*W*^*1118*^, UAS-*dZIP13* RNAi (v1362), UAS-*Malvolio* RNAi (v44000) and UAS-*Transferrin 1* RNAi (v106479) were sourced from the Vienna *Drosophila* RNAi Center (VDRC). UAS-*dZIP13* RNAi 1# (THU01588), UAS-*EZH2* RNAi (THU2831) and UAS-*TET* RNAi (TH04607.N) were acquired from Tsinghua Fly Center. Eyeless-Gal4 was purchased from Bloomington *Drosophila* Stock Center (BDSC). 10 × STAT92E-GFP (10 × STAT.GFP) [34], yw, ey-Flp; act > y^+^ Gal4 UAS-GFP; FRT82B tub-Gal80 [69] and w; Adv/Cyo; UAS-Raf^GOF^ FRT82B Scrib^−/−^/Tm6B (Raf^GOF^Scrib^−/−^) [69] were generously supplied by Dr. Tian Xu and Dr. Xianjue Ma. José Carlos Pastor-Pareja gratefully provided UAS-dome^ΔCYT2.1^ (UAS-dome^DN^) [65]. Upd3-LacZ [67] was gifted by Wei Song. The UAS-Malvolio overexpression (OE) [70] and UAS-Transferrin1 OE [39] have been previously described. The UAS-dZIP13 OE was constructed in our laboratory.

### Chemical treatments

All *Drosophila* were maintained at 25°C with approximately 60% relative humidity on standard cornmeal–yeast–agar medium. For chemical treatments, the following reagents and concentrations were used: 5 mM ferric ammonium citrate (FAC; Cat# F5879, Sigma–Aldrich), 100 μM bathophenanthroline disulfonic acid disodium salt (BPS; Cat# B1375, Sigma–Aldrich), and 200 μM rapamycin (Cat# 53123-88-9, MedChemExpress).

For rapamycin treatment, crosses were reared directly on food supplemented with 200 μM rapamycin. The drug was administered orally through the standard food medium at a concentration of 200 μM, based on previous studies and preliminary dose–response testing. A 1% DMSO solution (Cat# 67-68-5, MedChemExpress) was used as the vehicle control and added at the same volume as the drug. Larvae were exposed to the treatment from egg deposition until the late third-instar (L3) stage, which lasted approximately six days. Both vehicle-only and untreated control groups were maintained in parallel. After treatment, hemocyte number and proliferation were quantified 24 hours post-exposure.

### Bioinformatic analysis

Information regarding ZIP13 mRNA expression and clinical patient samples was sourced from The Cancer Genome Atlas (TCGA, http://cancergenome.nih.gov). The cBioPortal (http://www.cbioportal.org/) and the Kaplan-Meier Plotter web tool for Cancer Genomics (https://kmplot.com/analysis/) were utilized to assess the gene expression levels of hZIP13 in patients afflicted with various types of cancer.

### Survival assay

The survival assay was conducted following previously established methods [33,71]. We recorded the total number of individuals that perished during the larval stage (N1) or pupal stage (N2), as well as those that emerged as adults (N3). The relative survival rates (%) at various stages were calculated using these formulas: larval survival percentage (%) = 100% * (N1/ 50); pupal survival percentage (%) = 100% * (N2/ 50); adult emergence percentage (%) = 100% * (N3/ 50).

### Morphological analyses

Fluorescence in larvae, analysis of eye morphology, and examination of dissected tissues — including cephalic complexes, eye imaginal discs, and the ventral nerve cord (VNC) — were conducted 10 days post-oviposition [33]. In any given experimental setup, factors like exposure duration and fluorescence excitation intensity need to be consistent across all groups. Quantification of fluorescent intensity and tumor dimensions was carried out using ImageJ and analyzed with GraphPad prism 8.0. For each genotype, at least six flies were evaluated, with each experiment being replicated thrice.

### Ferrozine-based colorimetric assay

The ferrozine-based colourimetric assay was conducted following previously established protocols [44,72]. Approximately 150 cephalic complexes were harvested, washed three times with PBS, and then extracted using 0.1% PBST (Triton X-100) supplemented with 1% protease inhibitors. Protein concentrations were subsequently measured using the BCA kit (Cat#23227, Thermo Scientific). Afterwards, 22 µl of concentrated hydrochloric acid was added to the 100 µl sample, and the mixture was incubated at 95°C for 20 minutes, followed by centrifugation at 16,000 g. 75 mM ascorbic acid (36 µl) was added to the supernatant (90 µl) to reduce Fe^3+^ to Fe^2+^. Next, 10 mM ferrozine (36 µl, Cat#69898-45-9, Sangon Biotech) was added and mixed thoroughly.The saturated ammonium acetate (72 µl) was introduced to each group, and the absorbance was recorded at 562 nm. The iron standard curve (1–50 μM) was established and finally reported in μg Fe/mg protein. All experiments were conducted a minimum of three times.

### Aconitase activity assay

Aconitase activity was assessed according to the method outlined [39]. Briefly, ~ 150 cephalic complexes were dissected and extracted in 0.1% cold PBST with a 1% protease inhibitor cocktail. Protein concentrations from the extracts were equalized by the BCA method (Cat#23227, Thermo Scientific). Subsequently, 50 μg of the total protein sample was mixed with 700 μl of citrate reaction solution (50 mM K_2_HPO_4_, 30 mM citric acid, pH 7.4). Finally, the aconitase activity was measured in absorbance at OD_240_ every 20 seconds for 30 minutes at ambient temperature. The aconitate activity could be measured as difference between initial OD and the end OD at 30 mins. To analyze the data, pick two time points between which the rates are linearly increasing for all samples. Rate (OD/min) = (Absorbance 1 – Absorbance 2)/Time (min)*100%. The procedure was replicated thrice.

### TET activity assay

TET enzyme activity was determined using the Insect TET ELISA Assay Kit (Cat#JN77987X, Jining Shiye). Briefly, the samples (~50) were collected and centrifuged at 3000 × g at 4°C for 20 min. The concentration of the protein was measured utilizing the BCA assay kit (Cat#23227, Thermo Scientific). Next, 50 μl of standards or samples were introduced into the designated wells of the antibody-coated microtiter plate. After incubation at 37°C for 45 minutes, 50 μl of biotinylated anti-IgG and streptavidin-HRP was added to each well, followed by incubation at 37°C for 30 minutes. Then, 50 μl of the chromogen solutions A and B were mixed into the tubes. Repeat the above operation 4 times. TET activity was quantified by measuring absorbance at 450 nm following a 15-minute incubation at 37°C. The experiments were conducted a minimum of three times.

### Immunohistochemistry and fluorescence microscopy

The specified tissues were excised in cold phosphate-buffered saline (PBS), then fixed and washed with PBST. The following antibodies were utilized: anti-DE-Cadherin (1:100, Cat#DCAD2, Developmental Studies Hybridoma Bank (DSHB)), anti-beta-galactosidase (Cat#40-1a, DSHB), anti-PH3 (Cat#9701S, Cell signalling Technology), goat anti-rabbit IgG conjugated to Cy3 (1:500, Cat#BA1032, Boster), goat anti-mouse IgG conjugated to Cy3 (1:500, Cat#BA1031, Boster), and 4’,6-diamidino-2-phenylindole (DAPI, Cat#47165-04-8, MedChemExpress). Anti-NimC1 antibody (1:200, gift from Istvan Ando) [50] was used as a hemocyte marker. The specimens were secured in a medium composed of 50% glycerol mixed with PBS and visualized with a Zeiss LSM780 Meta confocal imaging system.

### Western blot analysis

Western blot analysis was conducted following previously established protocols [39]. ~ 50 cephalic complexes were homogenized directly in 150 µl of loading buffer (Cat#P0015L, Beyotime). The primary antibodies used were anti-EZH2 (1:500, PCRP-EZH2-1B3, DSHB), anti-DE-Cadherin (1:100, Cat#DCAD2, DSHB), anti-Tubulin (1:1000, Cat#ab56676, Abcam), anti-Histone H3 (1:1000, Cat#ab1791, Abcam), and anti-H3K27me3 (1:2000, Cat#139619, Cell signalling Technology). The secondary antibodies employed included goat anti-rabbit IgG (Cat#BA1054, Boster) and HRP-conjugated goat anti-mouse IgG (1:5000, Cat#BA1050, Boster).

### RNA isolation and quantitative real‑time PCR

A total of 20 wandering larvae or approximately 150 cephalic complexes were dissected and lysed with Trizol reagent (Cat#R401-01-AA, Vazyme) for RNA extraction. From 1.0 μg of the RNA samples, cDNA was synthesized using EasyTaq PCR SuperMix reagents (Catalog# AS111, TransGen Biotech). Quantitative analysis was performed with TransStart Green qPCR SuperMix reagents (Catalog# AQ101, TransGen Biotech) on a Roche LightCycler 96 system. The specific primer information used for amplifying upd1, upd2, upd3, EZH2 and chinmo is listed in Table 1.

**Table 1:** The primers used for qPCR.

Primer	Sequence
*rp49*-forward	AGGCCCAAGATCGTGAAGAA
*rp49*-reverse	TGTGCACCAGGAACTTCTTGA
upd1-forward	TCAGCTCAGCATCCCAATCAG
upd1-reverse	ATAGTCGATCCAGTTGCTGTTCCG
upd2-forward	TGCTATCGCTGAGGCTCTCG
upd2-reverse	GACTCTTCTCCGGCAAATCAGA
upd3-forward	CTGGTCACTGATCTTACTCGCC
upd3-reverse	GGATTGGTGGGATTGATGGGA
*chinmo*-forward	ACACCGAATACGCTGCTGGAG
*chinmo*-reverse	CACGCTGTTCTTGTTGTTCATCTTG
*EZH2*-forward	GGGGTAGTTGATGTTGACGC
*EZH2*-reverse	TGCCCTTGTCTGGAAAGTTAG

### Statistical analysis

Analysis of the data was conducted using Student’s *t*-test for pairwise comparisons and one-way ANOVA for comparisons among multiple groups. GraphPad (Prism 8.0 Software) was utilized to present statistical significance as mean ± SEM. Data quantification and analysis were carried out using ImageJ software. Asterisks denote the levels of statistical significance (*p < 0.05, **p < 0.01, ***p < 0.001).

## Supporting information

S1 Fig Overexpression of *dZIP13* enhances tumor growth and invasion in Raf^GOF^Scrib^−/−^ clones. **(A)** The boxplot shows ZIP13 expression across different cancers from the TNMplot.com Analysis Platform. The top, center, and bottom edges of the box represent the 75th, 50th (median), and 25th percentiles, respectively (*p < 0.05). **(B)** Representative confocal images of eye-antennal discs displaying GFP-labeled tumor clones expressing Raf^GOF^Scrib^−/−^ or Raf^GOF^Scrib^−/−^ with *dZIP13* overexpression (OE). GFP marks tumor clones. Scale bar: 200 μm. **(C)** Quantification of invasion frequency across tissues reveals enhanced invasion following *dZIP13* OE. **(D)** Quantification of tumor size shows that *dZIP13* OE significantly increases tumor burden compared to Raf^GOF^Scrib^−/−^ alone. Data are presented as mean ± SEM. Statistical significance was assessed using unpaired two-tailed Student′s *t*-test or chi-square test, as appropriate (**p < 0.01, ***p < 0.001). Genotypes: **(B-D)** *ey-Flp/ + ; Act > y*^*+*^*-Gal4, UAS-GFP/ +* ; FRT82B *tub-Gal80/UAS-Raf*^*GOF*^ FRT82B *Scrib*^−/−^ and *ey-Flp/ + ; Act > y*^*+*^*-Gal4, UAS-GFP/dZIP13 OE*; FRT82B *tub-Gal80/UAS-Raf*^*GOF*^ *FRT82B Scrib*^−/−^.
(TIF)

S2 Fig Iron accumulation promotes tumor growth via JAK/STAT signaling. **(A)** *dZIP13* RNAi or 1 mM FAC activates JAK/STAT signalling, while 100 μM BPS represses it. Scale bar: 100 μm. "NF" refers to normal food, used as the control diet in experiments. **(B)** Quantification of STAT.GFP intensity across genotypes (*n* = 6). **(C)** Survival rate of Raf^GOF^Scrib^−/−^ and *dZIP13* RNAi; Raf^GOF^Scrib^−/−^ larvae was rescued by blocking JAK/STAT signalling (*n* = 50 larvae per vial, *n* = 6 vials per experimental group). **(D)** Aconitase activity was induced in *dZIP13* RNAi, *Mvl* OE, or *Tsf1* OE, and suppressed by *Mvl* RNAi or *Tsf1* RNAi in *dZIP13* RNAi (*n* = 150 cephalic complexes per group). Data are presented as mean ± SEM. Statistical significance was calculated using unpaired two-tailed Student′s *t*-test (**p < 0.01, ***p < 0.001). Genotypes: **(A-B)** *ey-gal4/ + ; 10 × STAT.GFP/+* (control) and *ey-gal4/dZIP13 RNAi*; *10 × STAT.GFP/ +* . **(C)** *ey-Flp/ + ; Act > y*^*+*^*-Gal4, UAS-GFP/ +* ; FRT82B *tub-Gal80/UAS-Raf*^*GOF*^ FRT82B *Scrib*^*−/−*^, *ey-Flp/ + ; Act > y*^*+*^*-Gal4, UAS-GFP/dZIP13 RNAi*; FRT82B *tub-Gal80/UAS-Raf*^*GOF*^ *FRT82B Scrib*^*−/−*^, *ey-Flp/ + ; Act > y*^*+*^*-Gal4, UAS-GFP/UAS-Dome*^*DN*^; FRT82B *tub-Gal80/UAS-Raf*^*GOF*^ FRT82B *Scrib*^*−/−*^ and *ey-Flp/ + ; Act > y*^*+*^*-Gal4, UAS-GFP/UAS-Dome*^*DN*^*,dZIP13 RNAi*; FRT82B *tub-Gal80/UAS-Raf*^*GOF*^ *FRT82B Scrib*^*−/−*^. **(D)** *ey-gal4/ + ; 10 × STAT.GFP/+* (control), *ey-gal4/dZIP13 RNAi*; *10 × STAT.GFP/ +* , *ey-gal4/Mvl OE; 10 × STAT.GFP/ +* , *ey-gal4/UAS-Tsf1 OE*; *10 × STAT.GFP/ +* , *ey-gal4/dZIP13 RNAi; 10 × STAT.GFP/Mvl RNAi* and *ey-gal4/dZIP13 RNAi; 10 × STAT.GFP/Tsf1-RNAi*.
(TIF)

S3 Fig Iron regulates EZH2 expression in wild-type *Drosophila.* **(A)** Western blot analysis shows that iron regulates EZH2 expression under normal (non-tumor) conditions. **(B)** Quantification of EZH2 protein levels. Data are presented as mean ± SEM. Statistical significance was calculated using unpaired two-tailed Student′s *t*-test (*p < 0.05, ***p < 0.001).
(TIF)

S4 Fig The effect of iron on tumor invasion and migration was suppressed by *EZH2* knockdown. **(A)** The 3D structure of the EZH2 protein. **(B)** Chinmo mRNA levels in tumors were significantly reduced by BPS and dramatically induced by FAC. The regulation of iron on chinmo expression was suppressed by *EZH2* RNAi (*n* = 150 cephalic complexes per group). **(C-D)** The regulation of iron on tumor invasion **(C)** and migration **(D)** was absent in *EZH2* RNAi. (*n* = 30 animals from three independent experiments). Data are presented as mean ± SEM. Statistical significance was calculated using unpaired two-tailed *t*-test or one-way ANOVA (*p < 0.05, **p < 0.01, ns no significant). Genotypes: **(B-D)** *ey-Flp/ + ; Act > y*^*+*^*-Gal4, UAS-GFP/ +* ; FRT82B *tub-Gal80/UAS-Raf*^*GOF*^ FRT82B *Scrib*^*−/−*^ and *ey-Flp/ + ; Act > y*^*+*^*-Gal4, UAS-GFP/EZH2 RNAi*; FRT82B *tub-Gal80/UAS-Raf*^*GOF*^ *FRT82B Scrib*^*−/−*^.
(TIF)

S5 Fig Knockdown of *upd3* suppresses tumor growth and invasion in *dZIP13* RNAi; Raf^GOF^Scrib^−/−^ clones. **(A)** Eye-antennal discs containing *dZIP13* RNAi; Raf^GOF^Scrib^−/−^ clones (marked by GFP) with or without *upd3* RNAi expression. Tumor size and invasion were assessed by GFP signal. Clones with *upd3* RNAi showed significantly reduced overgrowth and decreased invasion into surrounding tissues. **(B-C)** Quantification of tumor area **(B)** and invasion frequency **(C)**. Data are presented as mean ± SEM from at least three independent experiments. Statistical significance was determined using unpaired two-tailed *t*-test or one-way ANOVA (***p < 0.001). Genotypes: **(A-C)** *ey-Flp/ + ; Act > y*^*+*^*-Gal4, UAS-GFP/ +* ; FRT82B *tub-Gal80/UAS-Raf*^*GOF*^ FRT82B *Scrib*^*−/−*^, *ey-Flp/ + ; Act > y*^*+*^*-Gal4, UAS-GFP/d*ZIP*13 RNAi*; FRT82B *tub-Gal80/UAS-Raf*^*GOF*^ FRT82B *Scrib*^*−/−*^, *ey-Flp/ + ; Act > y*^*+*^*-Gal4, UAS-GFP/upd3 RNAi*; FRT82B *tub-Gal80/UAS-Raf*^*GOF*^ FRT82B *Scrib*^*−/−*^ and *ey-Flp/ + ; Act > y*^*+*^*-Gal4, UAS-GFP/ upd3 RNAi,d*ZIP*13 RNAi*; FRT82B *tub-Gal80/UAS-Raf*^*GOF*^ *FRT82B Scrib*^*−/−*^.
(TIF)

S6 Fig Rapamycin inhibits hemocyte proliferation in malignant tumors. **(A–B)** Rapamycin treatment reduced the number of hemocytes **(A)** and the ratio of PH3-positive hemocytes to the total hemocyte population **(B)** adhering to the surface of the eye-antennal discs in tumors. Scale bar: 200 μm. Data are presented as mean ± SEM from at least three independent experiments. Statistical significance was determined using an unpaired two-tailed Student′s *t*-test or one-way ANOVA (*p < 0.05). Genotypes: **(A-B)** *ey-Flp/ + ; Act > y*^*+*^*-Gal4, UAS-GFP/ +* ; FRT82B *tub-Gal80/UAS-Raf*^*GOF*^ FRT82B *Scrib*^*−/−*^.
(TIF)

S7 Fig EZH2 regulates *upd* mRNA expression in malignant tumors. **(A–C)** Relative *upd1, upd2, and upd3* mRNA levels in tumors were significantly decreased by BPS treatment and strongly increased by FAC treatment. The iron-dependent regulation of *upd* expression was abolished by *EZH2* RNAi (n = 150 cephalic complexes per group). Data are presented as mean ± SEM from at least three independent experiments. Statistical significance was determined using an unpaired two-tailed Student′s *t*-test or one-way ANOVA (**p < 0.01, **p < 0.001; ns, not significant). Genotypes: **(A-C)** *ey-Flp/ + ; Act > y*^*+*^*-Gal4, UAS-GFP/ +* ; FRT82B *tub-Gal80/UAS-Raf*^*GOF*^ FRT82B *Scrib*^*−/−*^ and *ey-Flp/ + ; Act > y*^*+*^*-Gal4, UAS-GFP/EZH2 RNAi*; FRT82B *tub-Gal80/UAS-Raf*^*GOF*^ *FRT82B Scrib*^*−/−*^.
(TIF)
