# Supplementary figures and images for: Genetic dissection of signalling pathways that mediate iron-related tumor growth in a Drosophila model
Source: PLoS Genet. 2026 Feb 13;22(2):e1012017. doi: 10.1371/journal.pgen.1012017 (PMC12904468; doi:10.1371/journal.pgen.1012017)

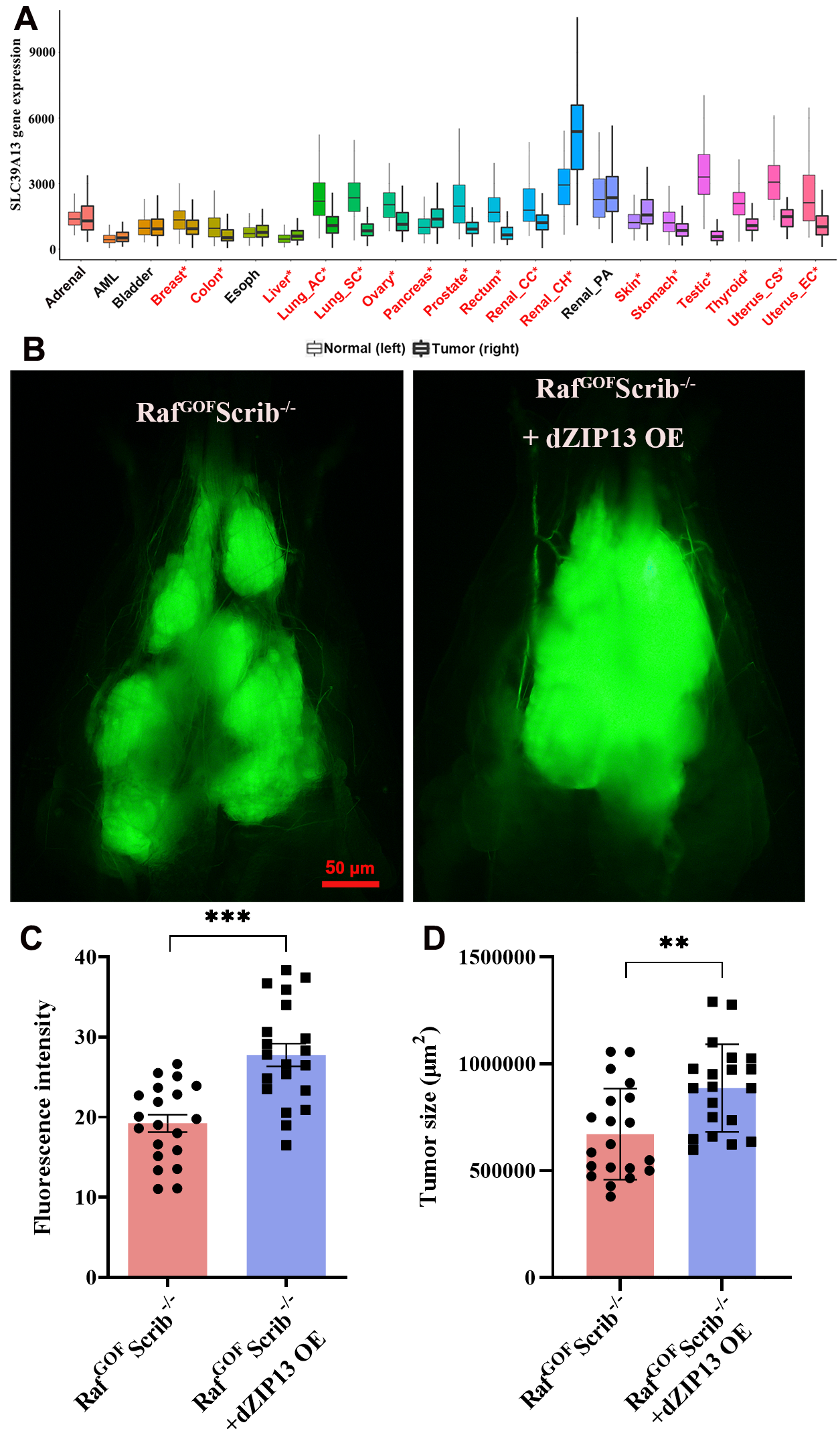

Supplement: S1 Fig — (A) The boxplot shows ZIP13 expression across different cancers from the TNMplot.com Analysis Platform. The top, center, and bottom edges of the box represent the 75th, 50th (median), and 25th percentiles, respectively (*p < 0.05). (B) Representative confocal images of eye-antennal discs displaying GFP-labeled tumor clones expressing RafGOFScrib−/− or RafGOFScrib−/− with dZIP13 overexpression (OE). GFP marks tumor clones. Scale bar: 200 μm. (C) Quantification of invasion frequency across tissues reveals enhanced invasion following dZIP13 OE. (D) Quantification of tumor size shows that dZIP13 OE significantly increases tumor burden compared to RafGOFScrib−/− alone. Data are presented as mean ± SEM. Statistical significance was assessed using unpaired two-tailed Student′s t-test or chi-square test, as appropriate (**p < 0.01, ***p < 0.001). Genotypes: (B-D) ey-Flp/ + ; Act > y+-Gal4, UAS-GFP/ + ; FRT82B tub-Gal80/UAS-RafGOF FRT82B Scrib−/− and ey-Flp/ + ; Act > y+-Gal4, UAS-GFP/dZIP13 OE; FRT82B tub-Gal80/UAS-RafGOF FRT82B Scrib−/−. (TIF) [file pgen.1012017.s001.tif]

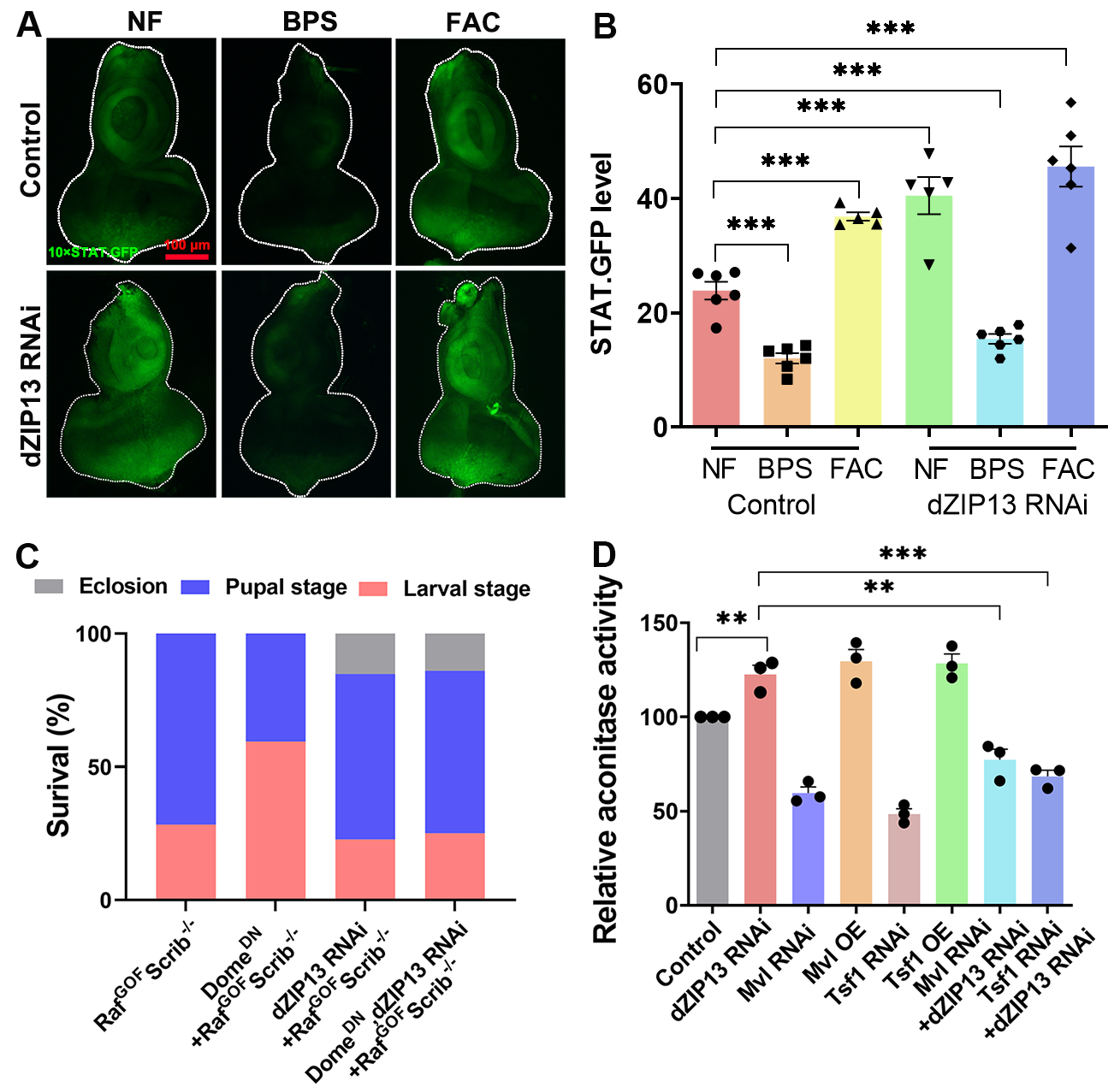

Supplement: S2 Fig — (A) dZIP13 RNAi or 1 mM FAC activates JAK/STAT signalling, while 100 μM BPS represses it. Scale bar: 100 μm. "NF" refers to normal food, used as the control diet in experiments. (B) Quantification of STAT.GFP intensity across genotypes (n = 6). (C) Survival rate of RafGOFScrib−/− and dZIP13 RNAi; RafGOFScrib−/− larvae was rescued by blocking JAK/STAT signalling (n = 50 larvae per vial, n = 6 vials per experimental group). (D) Aconitase activity was induced in dZIP13 RNAi, Mvl OE, or Tsf1 OE, and suppressed by Mvl RNAi or Tsf1 RNAi in dZIP13 RNAi (n = 150 cephalic complexes per group). Data are presented as mean ± SEM. Statistical significance was calculated using unpaired two-tailed Student′s t-test (**p < 0.01, ***p < 0.001). Genotypes: (A-B) ey-gal4/ + ; 10 × STAT.GFP/+ (control) and ey-gal4/dZIP13 RNAi; 10 × STAT.GFP/ + . (C) ey-Flp/ + ; Act > y+-Gal4, UAS-GFP/ + ; FRT82B tub-Gal80/UAS-RafGOF FRT82B Scrib−/−, ey-Flp/ + ; Act > y+-Gal4, UAS-GFP/dZIP13 RNAi; FRT82B tub-Gal80/UAS-RafGOF FRT82B Scrib−/−, ey-Flp/ + ; Act > y+-Gal4, UAS-GFP/UAS-DomeDN; FRT82B tub-Gal80/UAS-RafGOF FRT82B Scrib−/− and ey-Flp/ + ; Act > y+-Gal4, UAS-GFP/UAS-DomeDN,dZIP13 RNAi; FRT82B tub-Gal80/UAS-RafGOF FRT82B Scrib−/−. (D) ey-gal4/ + ; 10 × STAT.GFP/+ (control), ey-gal4/dZIP13 RNAi; 10 × STAT.GFP/ + , ey-gal4/Mvl OE; 10 × STAT.GFP/ + , ey-gal4/UAS-Tsf1 OE; 10 × STAT.GFP/ + , ey-gal4/dZIP13 RNAi; 10 × STAT.GFP/Mvl RNAi and ey-gal4/dZIP13 RNAi; 10 × STAT.GFP/Tsf1-RNAi. (TIF) [file pgen.1012017.s002.tif]

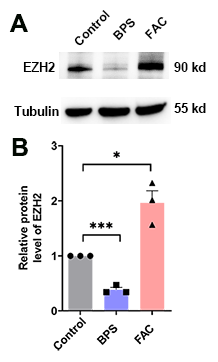

Supplement: S3 Fig — (A) Western blot analysis shows that iron regulates EZH2 expression under normal (non-tumor) conditions. (B) Quantification of EZH2 protein levels. Data are presented as mean ± SEM. Statistical significance was calculated using unpaired two-tailed Student′s t-test (*p < 0.05, ***p < 0.001). (TIF) [file pgen.1012017.s003.tif]

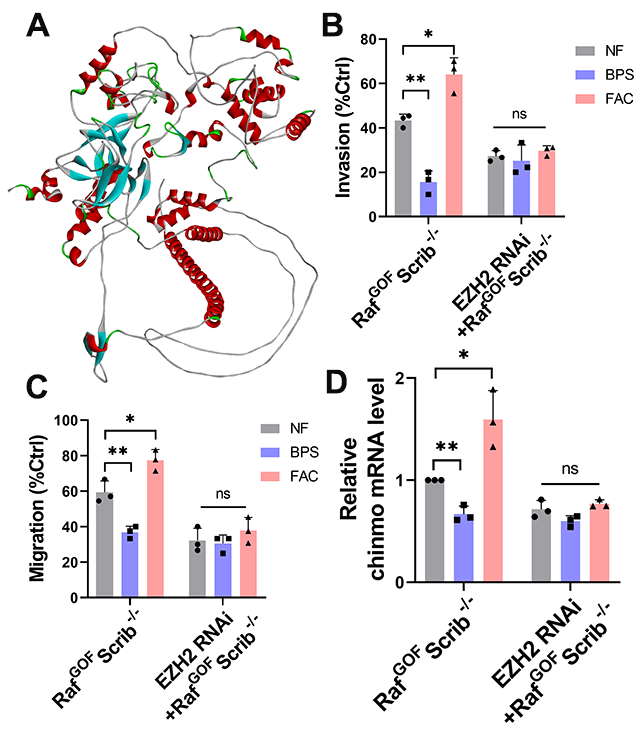

Supplement: S4 Fig — (A) The 3D structure of the EZH2 protein. (B) Chinmo mRNA levels in tumors were significantly reduced by BPS and dramatically induced by FAC. The regulation of iron on chinmo expression was suppressed by EZH2 RNAi (n = 150 cephalic complexes per group). (C-D) The regulation of iron on tumor invasion (C) and migration (D) was absent in EZH2 RNAi. (n = 30 animals from three independent experiments). Data are presented as mean ± SEM. Statistical significance was calculated using unpaired two-tailed t-test or one-way ANOVA (*p < 0.05, **p < 0.01, ns no significant). Genotypes: (B-D) ey-Flp/ + ; Act > y+-Gal4, UAS-GFP/ + ; FRT82B tub-Gal80/UAS-RafGOF FRT82B Scrib−/− and ey-Flp/ + ; Act > y+-Gal4, UAS-GFP/EZH2 RNAi; FRT82B tub-Gal80/UAS-RafGOF FRT82B Scrib−/−. (TIF) [file pgen.1012017.s004.tif]

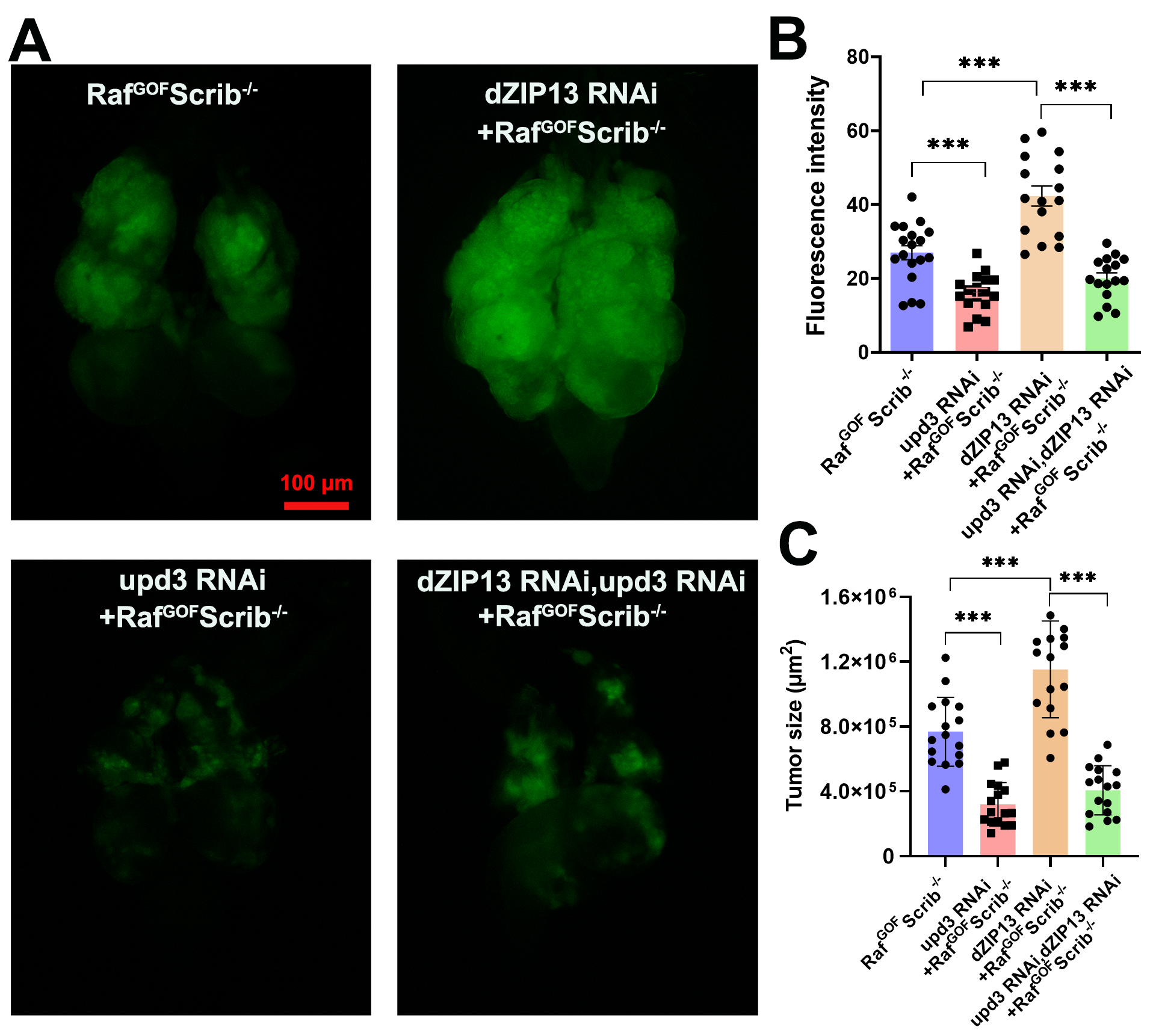

Supplement: S5 Fig — (A) Eye-antennal discs containing dZIP13 RNAi; RafGOFScrib−/− clones (marked by GFP) with or without upd3 RNAi expression. Tumor size and invasion were assessed by GFP signal. Clones with upd3 RNAi showed significantly reduced overgrowth and decreased invasion into surrounding tissues. (B-C) Quantification of tumor area (B) and invasion frequency (C). Data are presented as mean ± SEM from at least three independent experiments. Statistical significance was determined using unpaired two-tailed t-test or one-way ANOVA (***p < 0.001). Genotypes: (A-C) ey-Flp/ + ; Act > y+-Gal4, UAS-GFP/ + ; FRT82B tub-Gal80/UAS-RafGOF FRT82B Scrib−/−, ey-Flp/ + ; Act > y+-Gal4, UAS-GFP/dZIP13 RNAi; FRT82B tub-Gal80/UAS-RafGOF FRT82B Scrib−/−, ey-Flp/ + ; Act > y+-Gal4, UAS-GFP/upd3 RNAi; FRT82B tub-Gal80/UAS-RafGOF FRT82B Scrib−/− and ey-Flp/ + ; Act > y+-Gal4, UAS-GFP/ upd3 RNAi,dZIP13 RNAi; FRT82B tub-Gal80/UAS-RafGOF FRT82B Scrib−/−. (TIF) [file pgen.1012017.s005.tif]

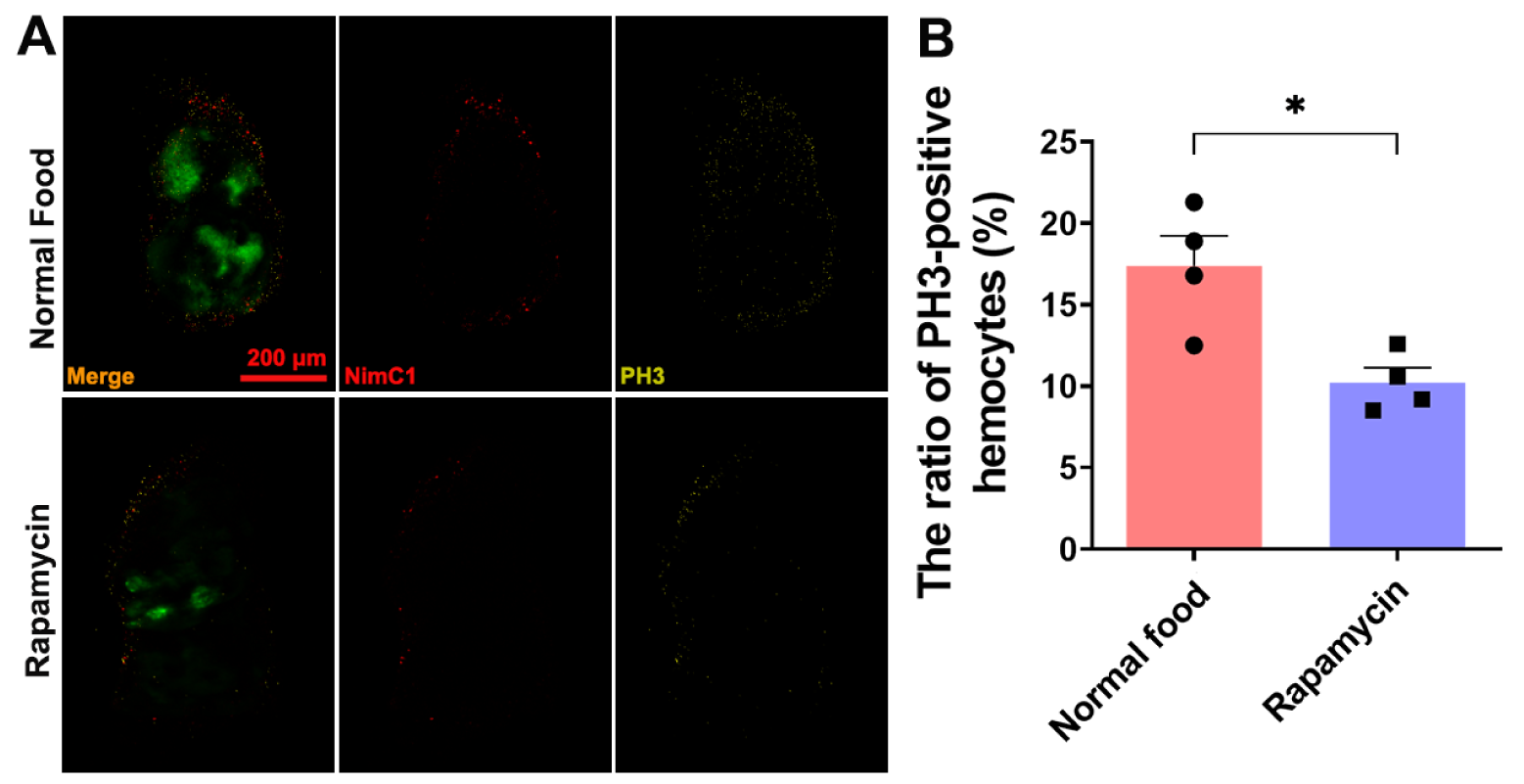

Supplement: S6 Fig — (A–B) Rapamycin treatment reduced the number of hemocytes (A) and the ratio of PH3-positive hemocytes to the total hemocyte population (B) adhering to the surface of the eye-antennal discs in tumors. Scale bar: 200 μm. Data are presented as mean ± SEM from at least three independent experiments. Statistical significance was determined using an unpaired two-tailed Student′s t-test or one-way ANOVA (*p < 0.05). Genotypes: (A-B) ey-Flp/ + ; Act > y+-Gal4, UAS-GFP/ + ; FRT82B tub-Gal80/UAS-RafGOF FRT82B Scrib−/−. (TIF) [file pgen.1012017.s006.tif]

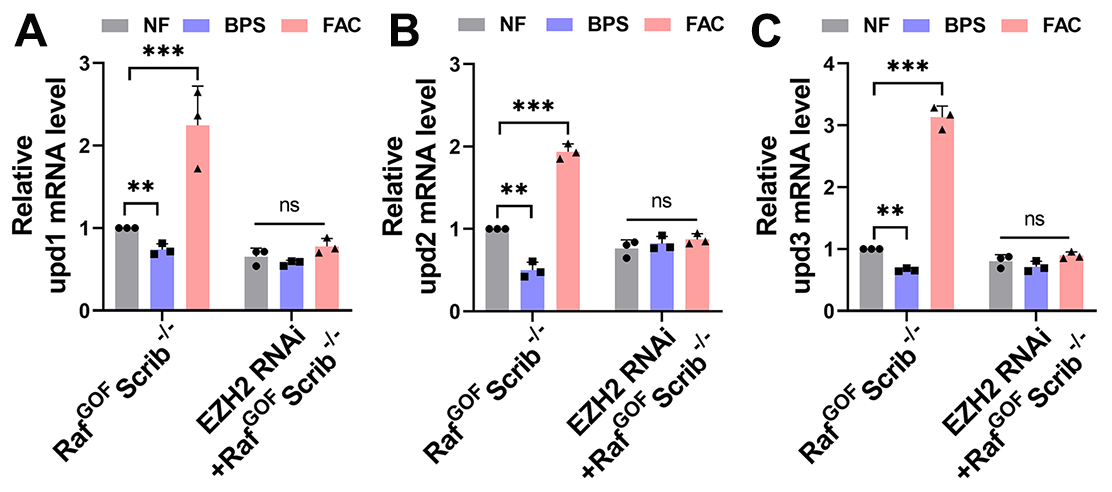

Supplement: S7 Fig — (A–C) Relative upd1, upd2, and upd3 mRNA levels in tumors were significantly decreased by BPS treatment and strongly increased by FAC treatment. The iron-dependent regulation of upd expression was abolished by EZH2 RNAi (n = 150 cephalic complexes per group). Data are presented as mean ± SEM from at least three independent experiments. Statistical significance was determined using an unpaired two-tailed Student′s t-test or one-way ANOVA (**p < 0.01, **p < 0.001; ns, not significant). Genotypes: (A-C) ey-Flp/ + ; Act > y+-Gal4, UAS-GFP/ + ; FRT82B tub-Gal80/UAS-RafGOF FRT82B Scrib−/− and ey-Flp/ + ; Act > y+-Gal4, UAS-GFP/EZH2 RNAi; FRT82B tub-Gal80/UAS-RafGOF FRT82B Scrib−/−. (TIF) [file pgen.1012017.s007.tif]
